# Supplementary material for: Molecular basis of foreign DNA recognition by BREX anti-phage immunity system
Source: Nat Commun. 2025 Feb 20;16:1825. doi: 10.1038/s41467-025-57006-2 (PMC11842806; doi:10.1038/s41467-025-57006-2)

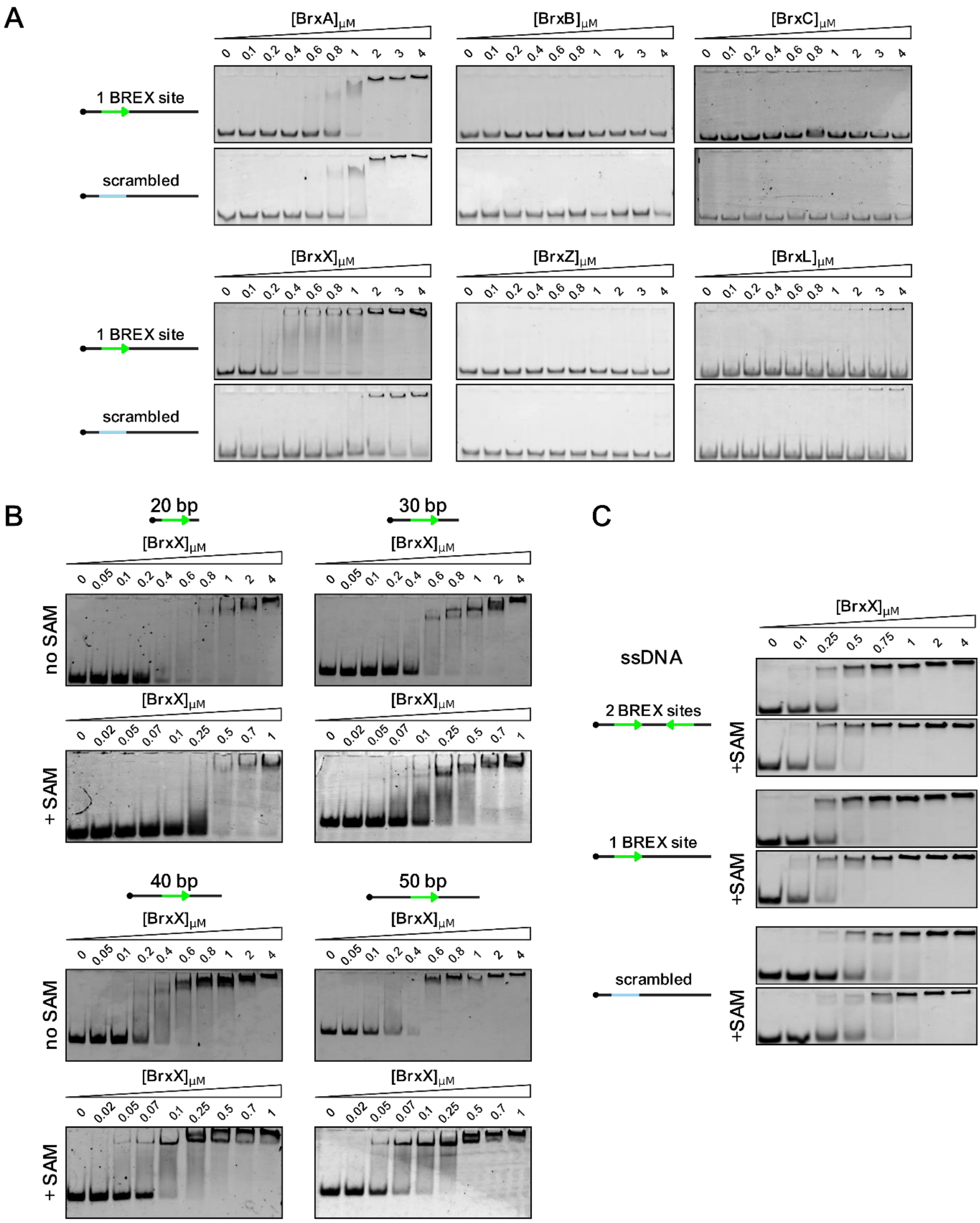


**Supplementary Figure S1. BREX proteins interact with DNA substrates.** (**A**) EMSA with 20 nM Cy5-labelled 43 bp dsDNA substrate without BREX sites (scrambled) or with one BREX site, incubated with indicated amount of BREX proteins. (**B**) EMSA with 20 nM Cy5-labelled dsDNA substrates of different length bearing one BREX site, incubated with indicated amount of BrxX without co-factors or in the presence of 0.5 mM SAM. (**C**) EMSA with 20 nM Cy5-labelled 43 bp ssDNA substrate without BREX sites (scrambled), with one, or two BREX sites, incubated with indicated amount of BrxX without co-factors or in the presence of 0.5 mM SAM. Representative gels from triplicate experiments are shown.

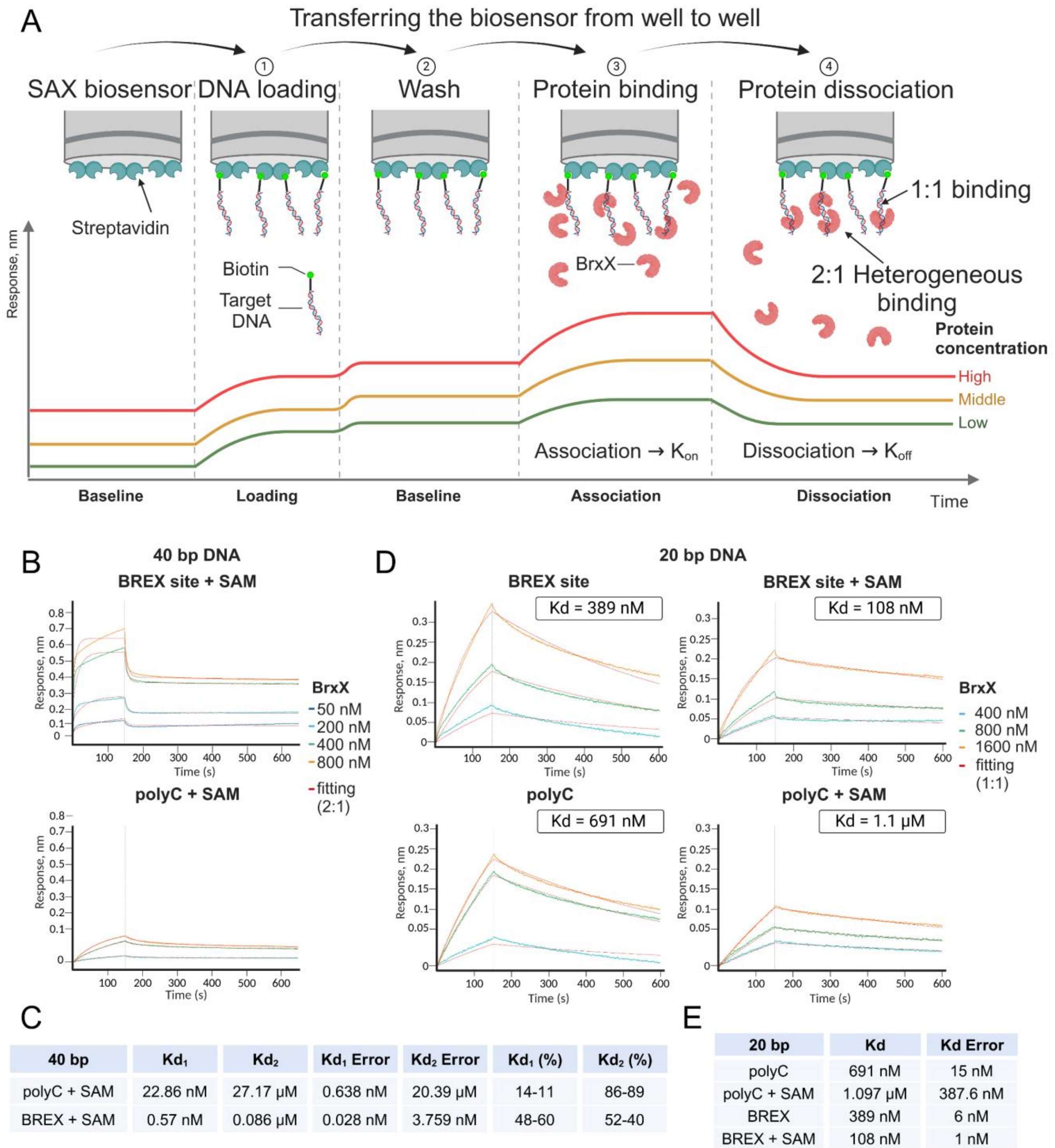


C

| 40 bp | Kd$_1$ | Kd$_2$ | Kd$_1$ Error | Kd$_2$ Error | Kd$_1$ (%) | Kd$_2$ (%) |
|---|---|---|---|---|---|---|
| polyC + SAM | 22.86 nM | 27.17 µM | 0.638 nM | 20.39 µM | 14-11 | 86-89 |
| BREX + SAM | 0.57 nM | 0.086 µM | 0.028 nM | 3.759 nM | 48-60 | 52-40 |

E

| 20 bp | Kd | Kd Error |
|---|---|---|
| polyC | 691 nM | 15 nM |
| polyC + SAM | 1.097 µM | 387.6 nM |
| BREX | 389 nM | 6 nM |
| BREX + SAM | 108 nM | 1 nM |

**Supplementary Figure S2. Biolayer Interferometry (BLI) measurement of BrxX:DNA interaction.** (**A**) A principle of the BLI assay, measuring BrxX interaction with biotinylated dsDNA substrate. Examples of kinetic curves are provided. Two binding modes (1:1 and 2:1) describing possible BrxX:DNA interactions are presented. (**B**) Association and dissociation kinetics of BrxX interacting with a 40 bp biotinylated dsDNA substrate bearing one BREX site or a polyC sequence. 0.5 mM SAM was present when indicated. Determined kinetic parameters are shown in (**C**) and the data was fit to the 2:1 binding model. (**D**) Association and dissociation kinetics of BrxX interacting with a 20 bp biotinylated dsDNA substrate bearing one BREX site or a polyC sequence. 0.5 mM SAM was present when indicated. Determined kinetic parameters are shown in (**E**) and the data was fit to the 1:1 binding model. Panel A of this supplementary figure was created with BioRender.com.

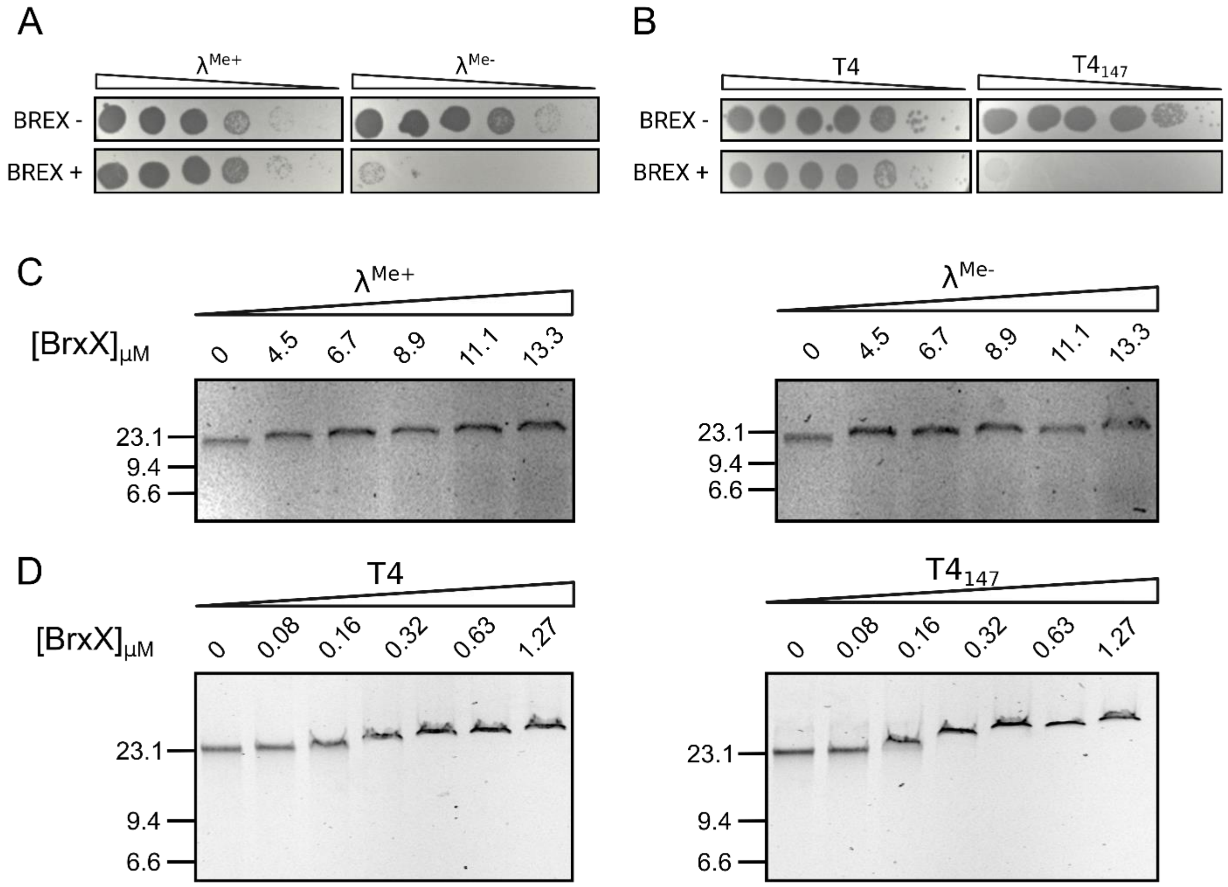


**Supplementary Figure S3. BrxX binds modified genomic DNA of T4 and λ phages.** EOP assay demonstrating BREX sensitivity to the $\lambda_{ts}$ phage induced from the BREX$^{+}$ lysogen ($\lambda^{Me+}$) (**A**) and to the WT T4 phage (**B**), in contrast to the non-modified $\lambda_{ts}$ ($\lambda^{Me-}$) or $T4_{147}$, lacking DNA glucosylation. (**C**) EMSA with BxrX and 130 pM genomic DNA of BREX-modified ($\lambda^{Me+}$) and non-modified ($\lambda^{Me-}$) phage. (**D**) EMSA with BxrX and 22 pM genomic DNA of WT glucosylated phage T4 or non-glycosylated genomic DNA of the phage $T4_{147}$.

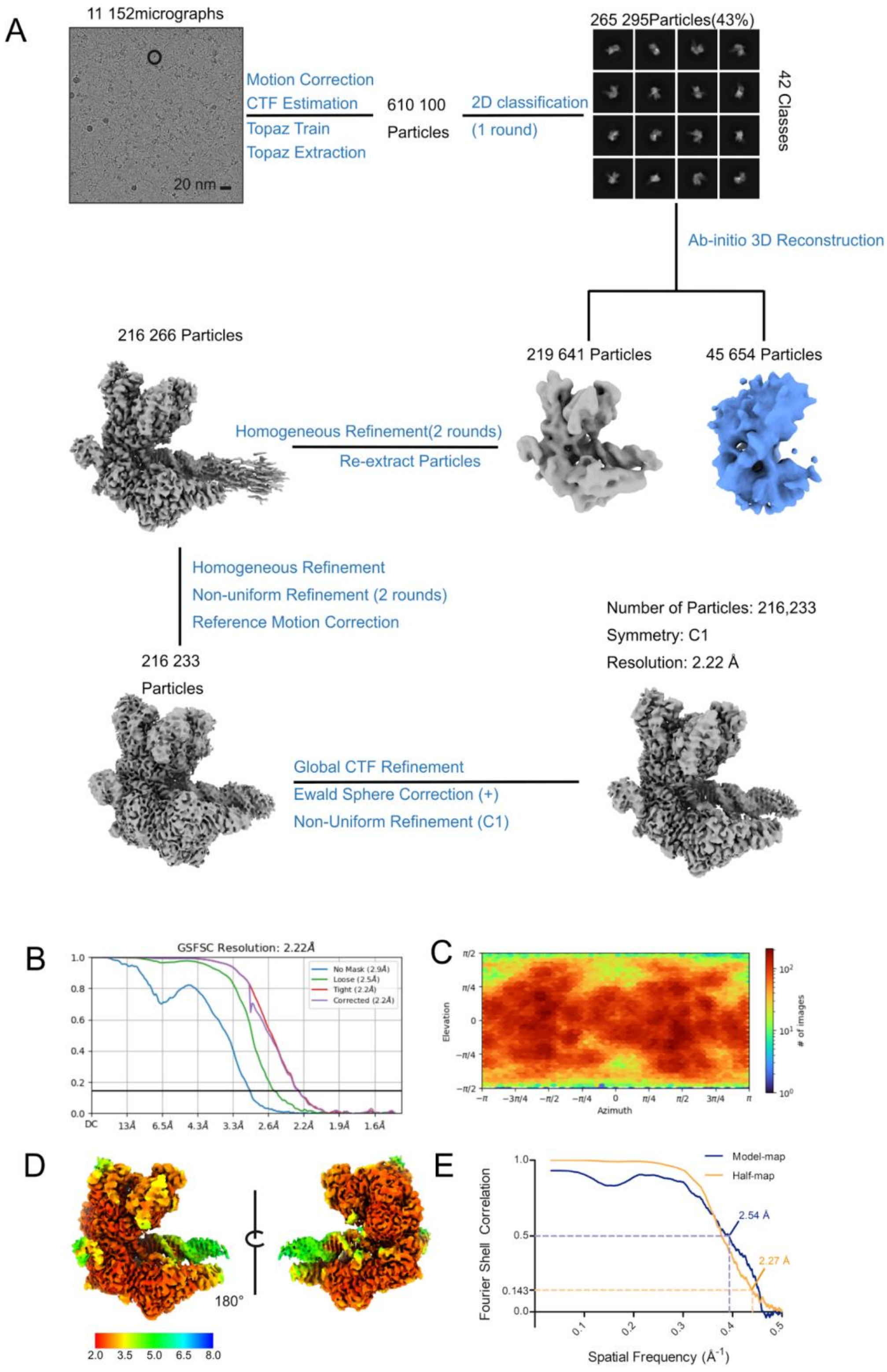


**Supplementary Figure S4. CryoEM data processing for BrxX:DNA.** (**A**) A representative motion-corrected micrograph and the processing scheme (see *Methods* for description). (**B**) FSC curve for the final reconstruction as output by cryoSPARC. (**C**) Euler angle distribution as output by cryoSPARC. (**D**) Local resolution maps illustrating resolution distribution. (**E**) Map-to-model FSC curve (blue) compared with half-maps FSC curve (orange) as output by Phenix.mtriage (soft mask based on the atomic model is used). FSC=0.5 (for map-to-model FSC) and FSC=0.143 (for half-map FSC) values are indicated with arrows.

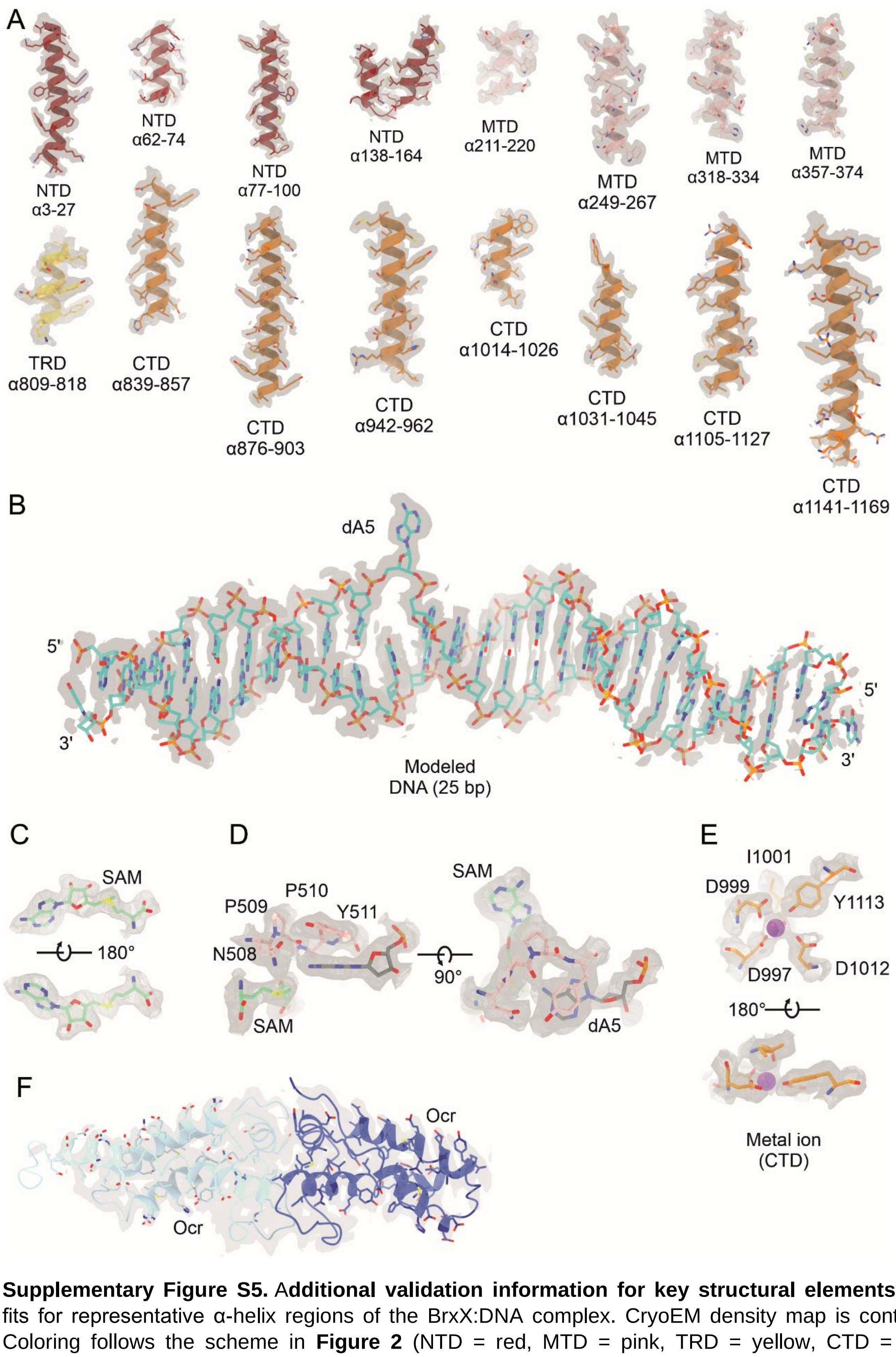


**Supplementary Figure S5. Additional validation information for key structural elements.** (**A**) Density fits for representative α-helix regions of the BrxX:DNA complex. CryoEM density map is contoured at 5σ. Coloring follows the scheme in **Figure 2** (NTD = red, MTD = pink, TRD = yellow, CTD = orange) and numbering represents first (always shown on the bottom or right of the depiction) and last amino acid displayed. (**B**) Density fit for DNA nucleotides bound to BrxX contoured at 5σ. (**C**) A Coulomb potential density map for S-adenosyl methionine (SAM) ligand contoured at 5σ. (**D**) Density fit for the binding site of the SAM molecule (both contoured at the same 5σ level). (**E**) Density fit for the binding site of a metal ion contoured to 5σ. (**F**) Density fit for the Ocr dimer; map contoured at 7σ.

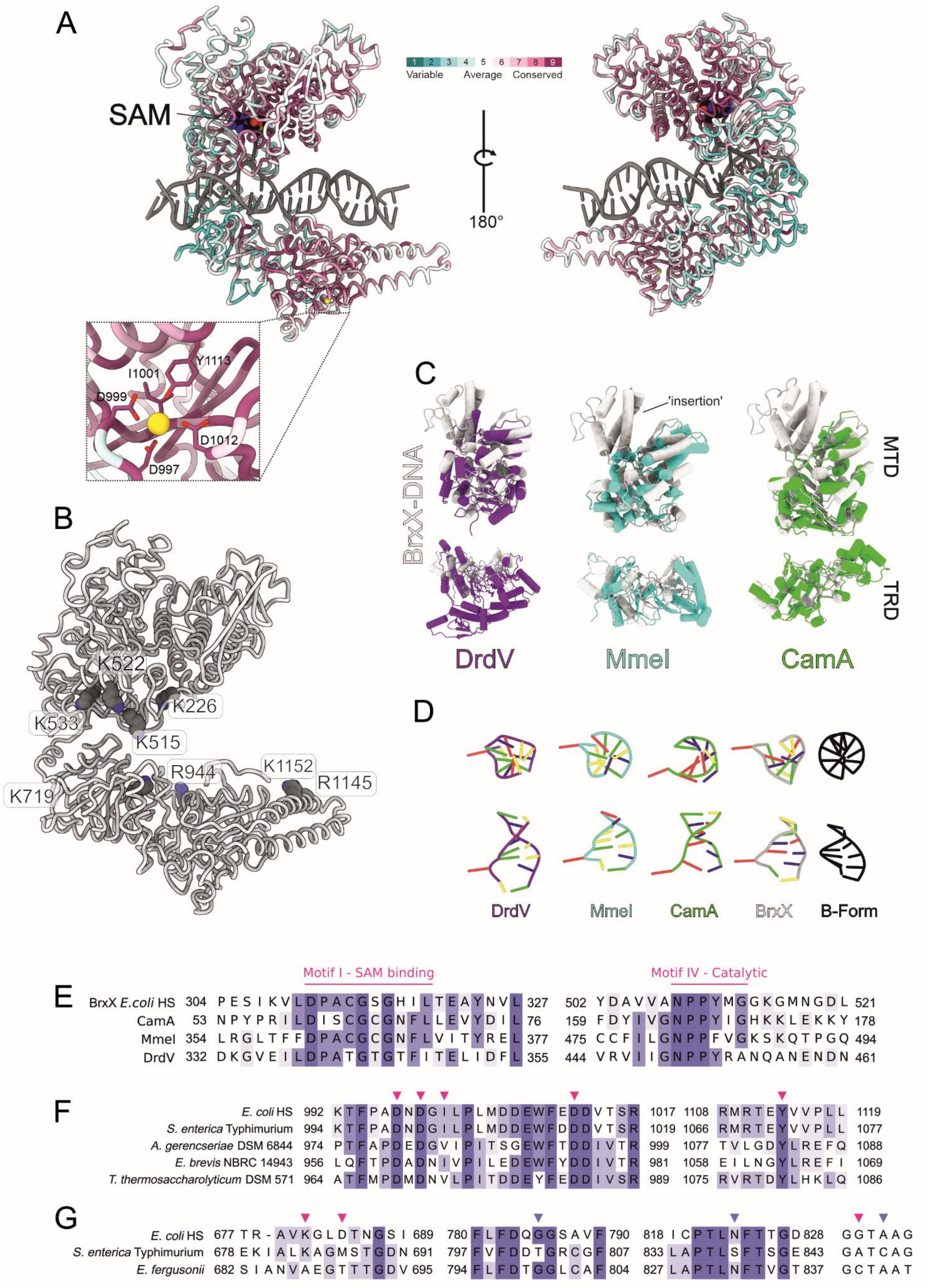
A
SAM
1 2 3 4 5 6 7 8 9
Variable Average Conserved
180°
I1001
Y1113
D999
D1012
D997
B
K522
K226
K533
K515
K1152
R944
R1145
K719
C
'insertion'
BrxX-DNA
MTD
TRD
DrdV
MmeI
CamA
D
DrdV
MmeI
CamA
BrxX
B-Form
Motif I - SAM binding
Motif IV - Catalytic
E
BrxX E.coli HS 304 PESIKVLDPACGSGHILTEAYNVL 327 502 YDAVVANPPYMGGKGMNGDL 521
CamA 53 NPYPRILDISCGCGNFLLEVYDIL 76 159 FDYIVGNPPYIGHKKLEKKY 178
MmeI 354 LRGLTFFDPACGCGNFLVITYREL 377 475 CCFILGNPPFVGKSKQTPGQ 494
DrdV 332 DKGVEILDPATGTGTFITELIDFL 355 444 VRVIIGNPPYRANQANENDN 461
F
E. coli HS 992 KTFPADNDGILPLMDDEWFEDDVTSR 1017 1108 RMRTEYVVPLL 1119
S. enterica Typhimurium 994 KTFPADNDGILPLMDDEWFDDDVTSR 1019 1066 RMRTEYVVPLL 1077
A. gerencseriae DSM 6844 974 PTFAPDEDGVIPITSGEWFTDDIVTR 999 1077 TVLGDYLREFQ 1088
E. brevis NBRC 14943 956 LQFTPDADNIVPILEDEWFYDDIVTR 981 1058 EILNGYLREFI 1069
T. thermosaccharolyticum DSM 571 964 ATFMPDMDNVLPITDDEYFEDDIVSR 989 1075 RVRTDYLHKLQ 1086
G
E. coli HS 677 TR-AVKGLDTNGSI 689 780 FLFDQGGSAVF 790 818 ICPTLNFTTGD 828 GGTAAG
S. enterica Typhimurium 678 EKIALKAGMSTGDN 691 797 FVFDDTGRCGF 807 833 LAPTLSFTSGE 843 GATCAG
E. fergusonii 682 SIANVAEGTTTGDV 695 794 FLFDTGGLCAF 804 827 LAPTLNFTVGT 837 GCTAAT

**Supplementary Figure S6. Conservation of BrxX domains and motifs in comparison with homologous MTases.** (**A**) A cartoon representation of a BrxX:DNA model colored by ConSurf1 conservation scores. SAM molecule and a metal ion (annotated as $Mg^{2+}$) are shown as van der Waals spheres. Note the high degree of conservation around the SAM-binding pocket and the meta-binding site (inset); TRD has a relatively low level of conservation as a fastest-evolving region. (**B**) Amino acid residues contributing to the positively charged upper and lower "jaws" of the BrxX monomer interacting with DNA backbone. (**C**) Structural alignments of isolated BrxX MTD (*top*) and TRD (*bottom*) domains (white) with homologous domains from restriction-modification systems DrdV (purple, PDB:7LO5), MmeI (cyan, PDB:5HR4) and an orphan methyltransferase CamA (green, PDB:7LT5). Alignment was done by matchmaker algorithm in ChimeraX2. (**D**) Comparison of the backbone conformation of DNA between BrxX and the above enzymes. BrxX-bound DNA is most closely resembling DNA in MmeI structure. Ideal B-form DNA is shown in black. (**E**) Alignment of SAM-binding (Motif I) and methyltransferase catalytic (Motif IV) motifs of BrxX from *E. coli* HS (P0DUF9), orphan methylase CamA (Q183J3), Type IIL R-M methylase-nuclease MmeI (B2MU09), and restriction-modification systems DrdV (A0A345IJ72). (**F**) Alignment of the BrxX homologs CTD regions responsible for the recognition of the metal ion. (**G**) Alignment of the regions of BrxX TRD responsible for the recognition of the second (red boxes) and fourth (blue boxes) base pair in BREX site from *E. coli* HS (P0DUF9), *S. typhimurium* (A0A5A8QD96), and *E. fergusonii* (B7L3T0). Positions for mutagenesis were selected based on the alignment of E. coli HS structures with AF2 models of other BrxX variants.

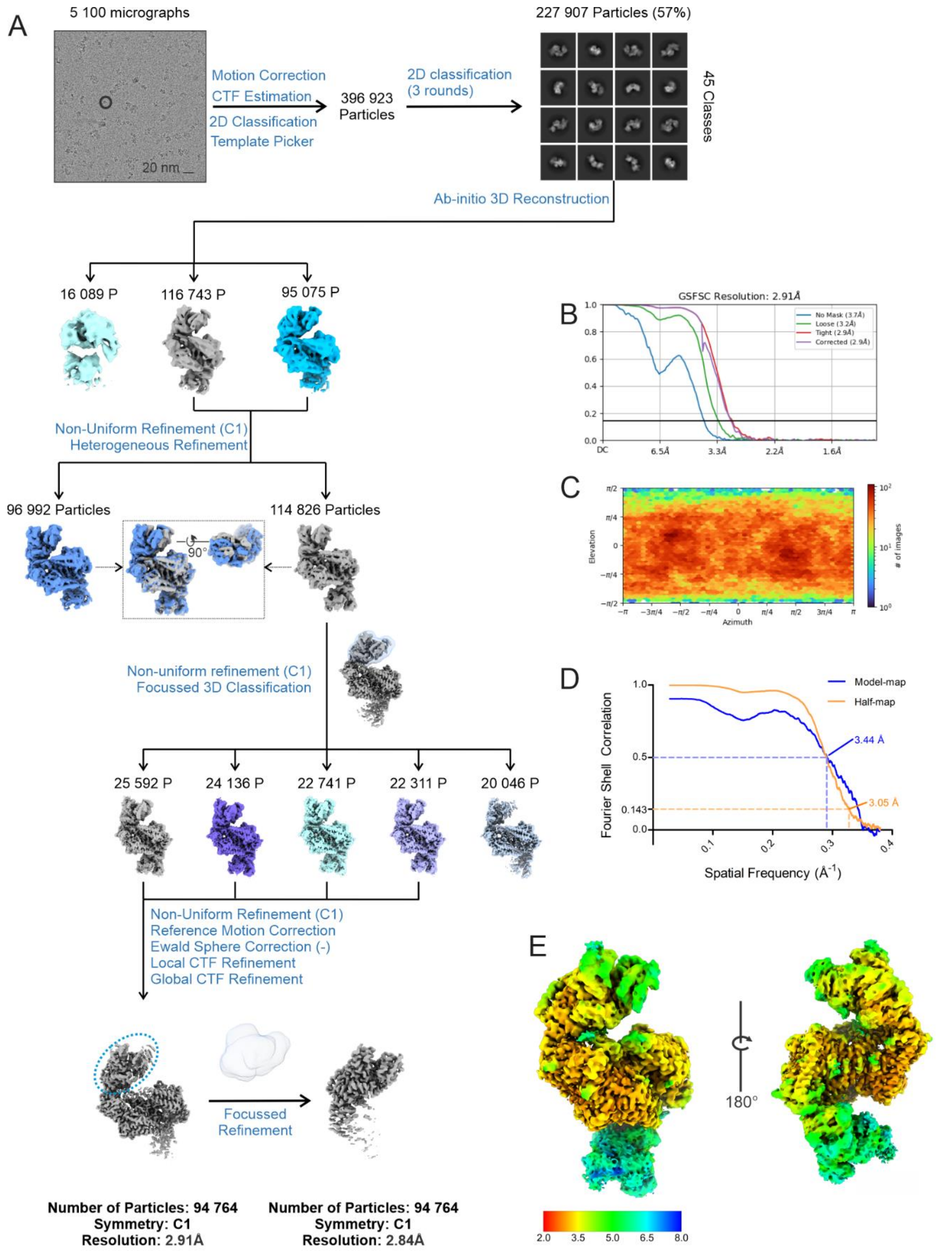


**Supplementary Figure S7. CryoEM data processing for BrxX:Ocr.** (**A**) A representative motion-corrected micrograph and the processing scheme (see *Methods* for description). (**B**) FSC curve for the final reconstruction as output by cryoSPARC. (**C**) Euler angle distribution as output by cryoSPARC. (**D**) Map-to-model FSC curve (blue) compared with half-maps FSC curve (orange) as output by Phenix.mtriage (soft mask based on the atomic model is used). FSC=0.5 (for map-to-model FSC) and FSC=0.143 (for half-map FSC) values are indicated with arrows. (**E**) Local resolution maps illustrating resolution distribution.

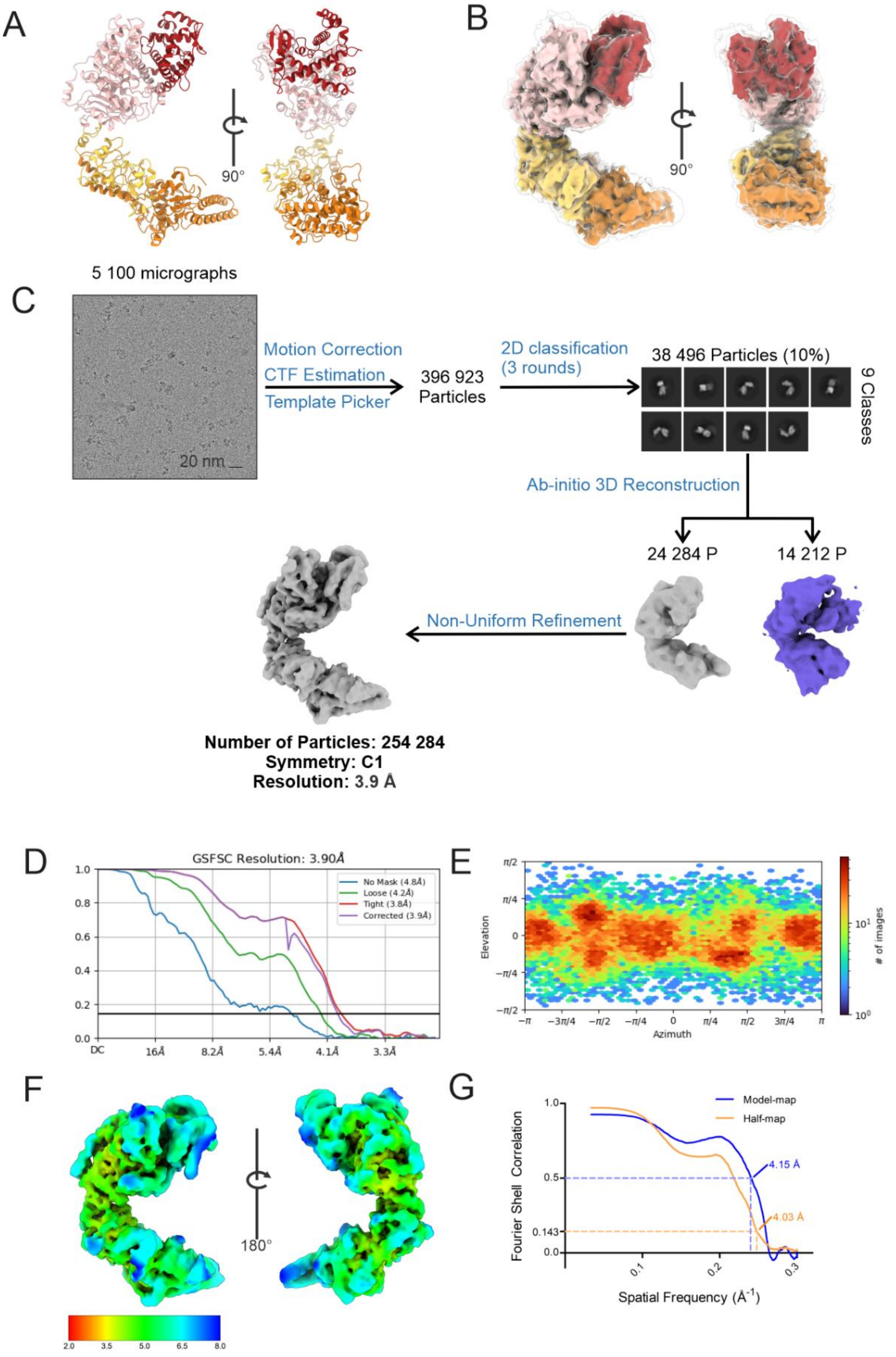


**Supplementary Figure S8. CryoEM data processing for BrxX$_{apo}$.** (**A**) A cartoon representation of the atomic model of Apo BrxX colored according to domains (NTD = red, MTD = pink, TRD = yellow, CTD = orange) as in **Figure 2**. (**B**) CryoEM density map of Apo BrxX. The autosharpened map is contoured at

7σ and shown within the transparent contour of the unsharpened map at 5σ. (**C**) A representative motion-corrected micrograph and the processing scheme (see *Methods* for description). (**D**) FSC curve for the final reconstruction as output by cryoSPARC. (**E**) Euler angle distribution as output by cryoSPARC. (**F**) Local resolution maps illustrating resolution distribution. (**G**) Map-to-model FSC curve (blue) compared with half-maps FSC curve (orange) as output by Phenix.mtriage (soft mask based on the atomic model is used). FSC=0.5 (for map-to-model FSC) and FSC=0.143 (for half-map FSC) values are indicated with arrows.

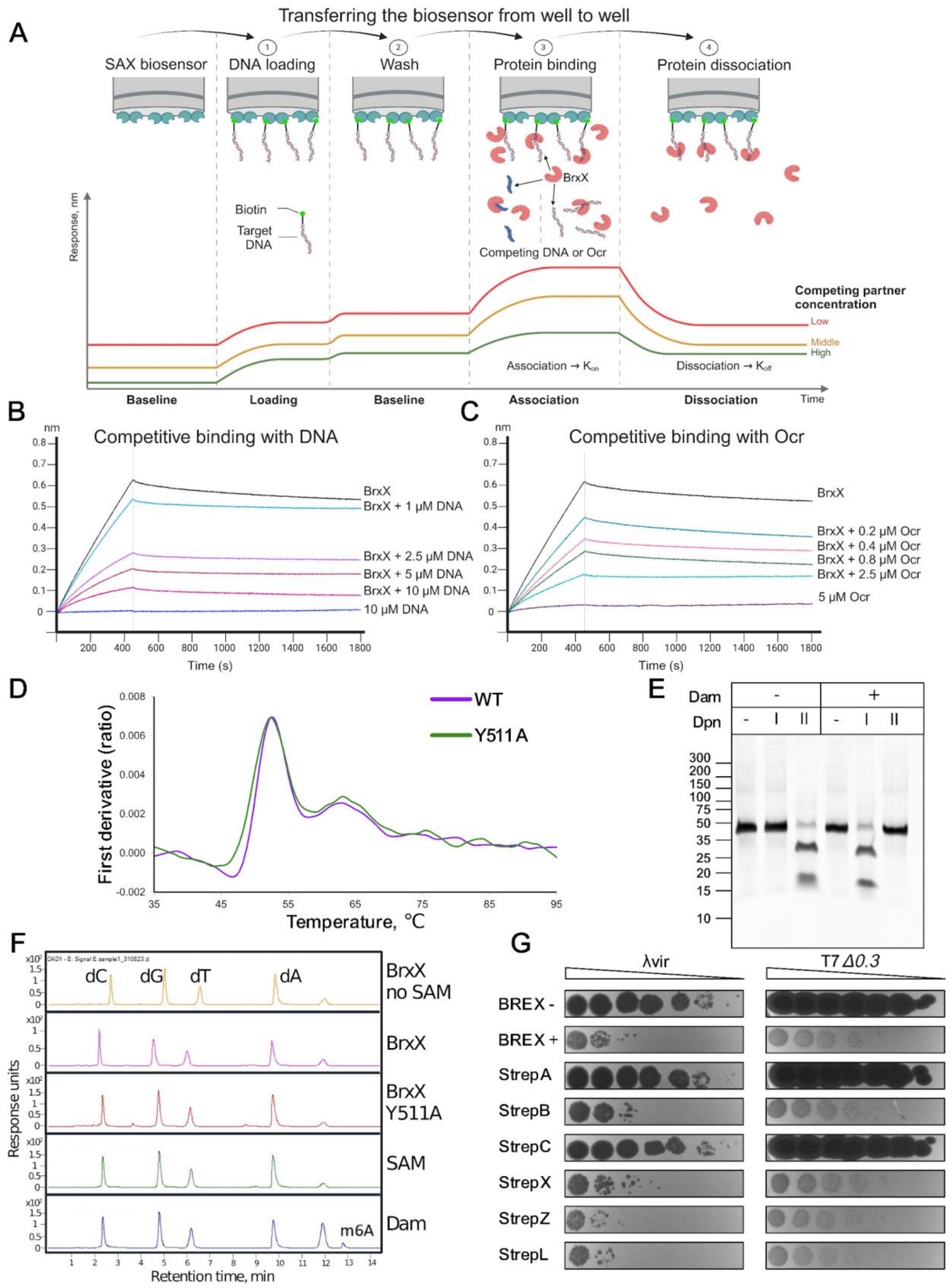


**Supplementary Figure S9. Additional supplementary information for Figures 5 and 6.** (**A**) A scheme of the BLI competitive binding assay designed to measure BrxX interaction with biotinylated DNA substrate in the presence of competing non-labeled DNA or Ocr. (**B,C**) Association and dissociation kinetics of 1.5 μM BrxX interacting with a 40 bp biotinylated dsDNA substate bearing one BREX site in the presence of a competing non-labelled dsDNA (**B**) or an Ocr dimer (**C**). (**D**) Thermal stability of BrxX and BrxX$^{Y511A}$ measured with Tycho NT.6. (**E**) DpnI/DpnII restriction-sensitivity assay with 50 bp dsDNA substrate containing a single Dam site and incubated with 100 μM Dam methyltransferase overnight. DpnI cleaves

only at methylated Dam sites, while DpnII cleaves only at non-methylated Dam sites. (**F**) HPLC-MS analysis of nucleosides obtained after digestion of a 40 bp dsDNA substrate, bearing an overlapping AluI/BREX site and a single Dam site, incubated with BrxX or Dam methyltransferase overnight. 0.5 mM SAM was present in the methylation reaction, unless otherwise indicated. SAM sample represents a control dsDNA substrate incubated in a SAM-containing buffer without a protein. Peak identity was validated with MS/MS analysis. (**G**) EOP analysis with BREX-sensitive phages $\lambda_{vir}$ and T7*Δ0.3* and BREX$^{-}$, BREX$^{+}$ and pBREX AL derivatives bearing Strep-tag (II) coding sequence at the 3’ end of each of the *brx* genes. Panel A of this supplementary figure was created with BioRender.com.

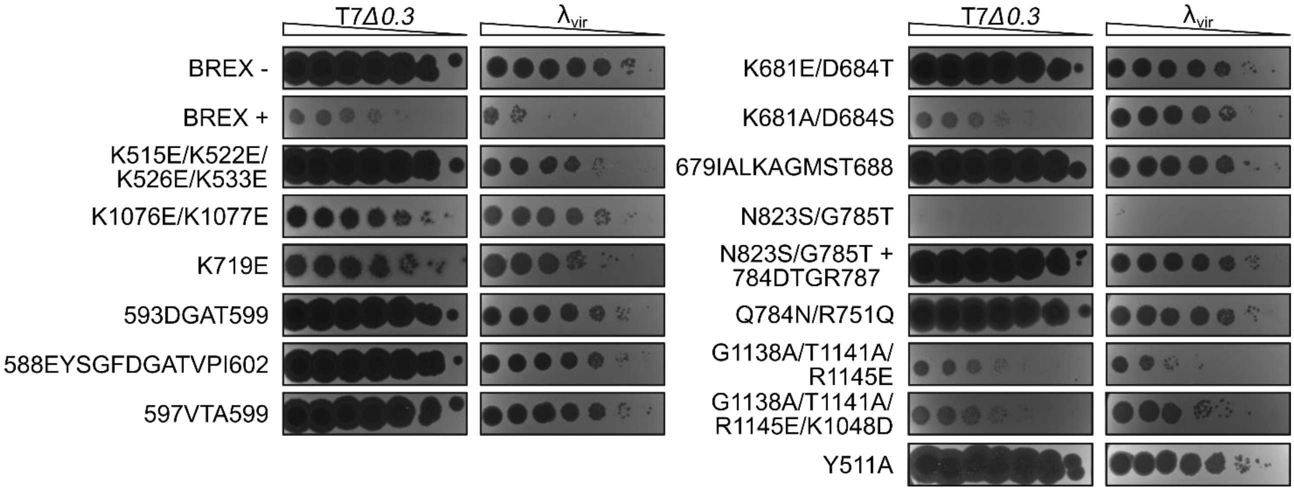


**Supplementary Figure S10. Summary of BrxX mutants constructed in this work.**

EOP analysis with BREX-sensitive phages $\lambda_{vir}$ and T7*Δ0.3* performed with BREX-, BREX+ and pBREX AL encoding BrxX with indicated mutations. Mutants' description is provided in Supplementary Table S1

**Supplementary Table S1. BrxX mutations obtained in this work and their effects**

| Predicted role of mutated residues | Mutation | Defence | DNA binding | Other features |
|---|---|---|---|---|
| Non-specific DNA binding (DNA backbone recognition) | K515E/K522E/K526E/K533E | - | - | |
| | K1076E/K1077E | - | | |
| | K719E | - | | |
| Substitution of the *E.coli* MTD loop recognizing the 5$^{th}$ base pair in BREX site with a predicted equivalent from *E. fergusonii,* different loop lengths were tested | 593(GQISGEV)599 to 593(DGAT)599 | - | | |
| | 588(GARAFGQISGEVVQT)602 to 588(EYSGFDGATVPI)602 | - | | |
| | 597(GEV)599 to 597(VTA)599 | - | | |
| Changing the specificity of BrxX TRD at the 2nd BREX site position G->C (*E. fergusonii* as reference) | K681E/D684T | - | | |
| Changing the specificity of BrxX TRD at the 2nd BREX site position G->A (*S. typhimurium* as reference) | K681A/D684S | + | | Modified BREX specificity |
| | 679IALKAGMST688 | - | | |
| Changing the specificity of BrxX TRD at the 4th BREX site position A->C (S. typhimurium as reference) | G785T/N823S | ++ | + | Modified BREX specificity |
| | N823S/G785T + 784DTGR787 | - | | |
| Changing the specificity of BrxX TRD at the 1st and the 3rd BREX site positions | Q784N/R751Q | - | | |
| Ocr binding | G1138A/T1141A/R1145E | + | | T7 phage sensitive |
| | G1138A/T1141A/R1145E/K1048D | + | +/- | T7 phage resistant, reduced DNA binding |
| Methyltransferase catalytic motif | Y511A | - | +/- | Non-specific DNA binding |

**Supplementary Table S2. Plasmids, bacteriophages and bacterial strains**

| Bacterial strains | Properties | Source |
|---|---|---|
| **BW25113** | *E. coli* K12 F– Δ(*araD-araB*)567 Δ*lacZ*4787(::*rrnB*-3) λ– *rph*-1 Δ(*rhaD-rhaB*)568 *hsdR*514 | Lab stock |
| **DH10B** | *E. coli F– mcrA Δ(mrr-hsdRMS-mcrBC) φ80lacZΔM15 ΔlacX74 recA1 endA1 araD139 Δ (ara-leu)7697 galU galK λ– rpsL($Str^R$) nupG* | Lab stock |
| **DH10B *galU*⁻** | DH10B, *galU*::KnR, for propagation of $T4_{147}$ phage | Lab stock |
| **$BREX^-$** | BW25113 carrying pBTB-2 | [3] |
| **$BREX^+$** | BW25113 carrying pBREXAL | [3] |
| **BW25113 $\lambda_{ts}$ lysogen** | $\lambda_{ts}$ lysogen of BW25113 | [3] |
| **BW25113 $\lambda_{wt}$ lysogen** | $\lambda_{wt}$ lysogen of BW25113 | This work |
| **QD5003** | Supports lysis by λ $cI_{857}$ $S_{am7}$ | Lab stock |
| **Bacteriophages** | | |
| **T7** | BREX resistant | Lab stock |
| **$T7_{\Delta 0.3}$** | *0.3::trxA*, BREX sensitive | [4] |
| **$T7_{fusion}$** | *0.3* and *0.7* fusion in a result of deletion (*Δ1256-2735*), leading to the production of semi-functional Ocr and increased BREX sensitivity | [4] |
| **T4** | BREX resistant, glycosylated 5-hmC in DNA | Lab stock |
| **$T4_{147}$** | BREX sensitive, 5-hmC-containing in DNA | [3] |
| **$\lambda_{vir}$** | λ mutant with an obligatory lytic lifecycle | Lab stock |
| **$\lambda_{ts}$** | $cI_{857}$ *bor::Cm* | Lab stock |
| **$\lambda_{Sam7}$** | $cI_{857}$ $S_{am7}$, does not support lysis of the cell | Lab stock |
| **Plasmids** | | |
| **pBTB-2** | pBBR1 origin, $Kan^R$ | Lab stock |
| **pBREX AL** | 6-genes E. coli HS BREX cluster cloned under native promoter in pBTB-2, $Kan^R$ | [3] |
| **pBAD-$His_6$-BrxN, N = A, B, C, X, Z, L** | pBAD-His/B, encoding BrxN protein with N-terminal $His_6$ tag, *araBAD* inducible promoter, $Amp^R$ | [3] |
| **pBAD-$His_6$-$BrxX^N$** | pBAD-His/B, encoding BrxX protein with N-terminal $His_6$ tag, N corresponds to one of the mutations mentioned in Table S1, *araBAD* inducible promoter, $Amp^R$ | This work |
| **pBAD-$His_6$-Ocr** | pBAD-His/B, encoding Ocr protein with N-terminal $His_6$ tag and TEV cleavage site, *araBAD* inducible promoter, $Amp^R$ | This work |
| **pBREX AL BrxN C-Strep, N=A,B,C, X, Z, L** | pBREX AL carrying BrxN with a Strep tag on the C-terminus, $Kan^R$ | [4] |
| **pBREX AL $BxrX^N$** | pBREX AL carrying BrxX with one of the mutations (N) provided in Table S1, $Kan^R$ | This work |
| **pBREX 1 ΔA** | pBTB-2 derivative expressing BrxB and BrxC proteins, *araBAD* inducible promoter, $Kan^R$ | [3] |
| **pBREX 2 ΔL** | pBAD derivative expressing BrxX and BrxZ proteins, *araBAD* inducible promoter, $Amp^R$ | [3] |
| **pBAD BrxX** | pBAD-His/B, encoding BrxX protein, *araBAD* inducible promoter, $Amp^R$ | [3] |
| **pHERD30t** | *E. coli/ - P. aeruginosa* shuttle vector, $Gnt^R$ | Gift of Prof. E. Westra |
| **pHERD30t_BREX/AluI** | pHERD30t with an insert of overlapping BREX/AluI sites, $Gnt^R$ | This work |

**Supplementary Table S3. List of oligonucleotides and DNA substrates used in the study**

| Name | Sequence (5’->3’) | Purpose |
|---|---|---|
| **BrxX mutagenesis** | | |
| **BrxX_Y511A_F_GA** | GAACCCGCCGGCTATGGGGGGGAAA | Construction of BrxX mutants in pBREX AL and pBAD context |
| **BrxX_Y511A_R_GA** | TTTCCCCCCCATAGCCGGCGGGTTC | |
| **BrxX_K681A/D684S_F_GA** | GCCGGACTAAGCACAAACGGGTCCATTGATGT | |
| **BrxX_K681A/D684S_R_GA** | CCGTTTGTGCTTAGTCCGGCCACAGCACGAGTTCCAAGAG | |
| **BrxX_N823S/G785T_F_GA** | CCTGGCCTGCATGAACTCTAAATATATCAACTACTCTGCAAGTTTAATTTGTCCTACGCTTAGCTTTACAACCGGTGATGTTAG | |
| **BrxX_N823S/G785T_R_GA** | TAGAGTTCATGCAGGCCAGGATGTTATATATCGATAGCTCATCATTATTTTCACAAAAAACAGCAGAACCGGTTTGGTCAAAAAGGAATCCTT | |
| **Brx_GA2_F** | TGAAACATGGCAAAGGTAGCGTTGC | |
| **Brx_GA2_R** | GCAACGCTACCTTTGCCATGTTTCA | |
| **BrxX_K515E/K522E/K526E/K533E_F_GA** | GAAGGGATGAATGGTGATCTGGAAGAGTTTGCTGAAAAACAATTCCCGGACAGCGAATCGGATTTGTTTGCGATGTT | |
| **BrxX_K515E/K522E/K526E/K533E_R_GA** | AGATCACCATTCATCCCTTCCCCCCCCATATACGGCGGGT | |
| **BrxX_K1076E/K1077E_F_GA** | GAAGAACGCCCAATCTACTGGCTATT | |
| **BrxX_K1076E/K1077E_R_GA** | CAGTAGATTGGGCGTTCTTCATACATCTTCATATGATCTT | |
| **BrxX_ K719E_F_GA** | GAAGGGGGTGAGTTGAGGAAGTG | |
| **BrxX_ K719E_R_GA** | TTCCTCAACTCACCCCCTTCTGCAATCGGAAACCATTTTG | |
| **BrxX_K681D/D684T_F_GA** | GATGGACTAACCACAAACGGGTCCATTGATGT | |
| **BrxX_K681D/D684T_R_GA** | CCGTTTGTGGTTAGTCCATCCACAGCACGAGTTCCAAGAG | |
| **BrxX_N823S/G785T_F_GA** | CCTGGCCTGCATGAACTCTAAATATATCAACTACTCTGCAAGTTTAATTTGTCCTACGCTTAGCTTTACAACCGGTGATGTTAG | |
| **BrxX_N823S/G785T_R_GA** | TAGAGTTCATGCAGGCCAGGATGTTATATATCGATAGCTCATCATTATTTTCACAAAAAACAGCAGAACCGGTTTGGTCAAAAAGGAATCCTT | |
| **BrxX_K1048D_F_GA** | GATCCGAAAAAAGGCGAATCTGC | |
| **BrxX_K1048D_R_GA** | GATTCGCCTTTTTTCGGATCGATCG | |

| | | |
|---|---|---|
| | CGTATAAACAGAGAC | |
| **BrxX_G1138A/T1141A/R1145E_F_GA** | GCGGAAGCCGCCCGCCTGAAAGAAGAACGCGACAGCCTGATCAA | |
| **BrxX_G1138A/T1141A/R1145E_R_GA** | TTCTTTCAGGCGGGCGGCTTCCGCGCCAGAAGCTTCATCAAGTT | |
| **X_ Y511A _F** | GAACCCGCCGGCGATGGG GGGGA | Construction of pBAD-His$_6$-BrxX$^{Y511A}$ |
| **X_ Y511A _R** | GCGACCACAGCATCATAAC | |
| **BrxX_Y511A_check_F** | GACGAAGCGGCGTTGAAA GC | |
| **Cloning of T7 *0.3*** | | |
| **AD7_N-His-TEV-Ocr_F** | TTTGGGCTAACAGGAGGAAGAATTCATGCATCATCATCATCATCATGAAAACCTGTATTTTCAGGGCGCTATGTCTAACATGACTTACA | Construction of pBAD-His$_6$-Ocr |
| **Ocr_R** | CAGCCAAGCTTGCGGCCGCGAGCTCTTACTCTTCATCCTCCTCGT | |
| **Methylation assay** | | |
| **AD31_pHERD_AluI_BREX_F** | CTTACCGCATACGCTACTTGCATTACAG | Construction of pHERD30t_BREX/AluI |
| **AD32_pHERD_AluI_BREX_R** | CTTACCGCTCACGCAACTGGTCC | |
| **AD33_40_BREX_Dam_AluI_For** | ACTCATCGGTACGATCGACGGTAAGCTTACCGGATAGGCA | dsDNA substrate for *in vitro* methylation |
| **AD34_40_BREX_Dam_AluI_Rev** | TGCCTATCCGGTAAGCTTACCGTCGATCGTACCGATGAGT | |
| **Probes for EMSA (BREX site is underlined)** | | |
| **AD10** | 5'Cy5-TCAGTAGTCAAGTCAGT<u>GGTAA$^{Me}$G</u>GTCAGGACAGGAGTCAG | 40 bp probe with methylated BREX site in the middle |
| **TRB2040** | 5'Cy5-TCAGTAGTCAAGTCAGT<u>GGTAAG</u>GTCAGGACAGGAGTCAG | 40 bp probe with BREX site in the middle |
| **TRB2039** | CTGACTCCTGTCCTGACCTTACCACTGACTTGACTACTGA | Reverse complement for AD10 and TRB2040 |
| **AD11_polyC** | 5'Cy5-TCAGTAGTCAAGTCAGT<u>CCCCCC</u>GTCAGGACAGGAGTCAG | 40 bp probe with polyC on place of BREX site |
| **AD12_polyC** | CTGACTCCTGTCCTGACGGGGGGACTGACTTGACTACTGA | |
| **AD72_4_T_for** | 5'Cy5-TCAGTAGTCAAGTCAGTGGTtAGGTCAGGACAGGAGTCAG | 40 bp EMSA probe with change in 4th position of BREX site |
| **AD73_4_T_rev** | CTGACTCCTGTCCTGACCTaACCACTGACTTGACTACTGA | |
| **AD74_4_C_for** | 5'Cy5-TCAGTAGTCAAGTCAGTGGTcAGGTCAGGACAGGAGTCAG | 40 bp EMSA probe with change in 4th position of BREX site |
| **AD75_4_C_rev** | CTGACTCCTGTCCTGACCTgACCACTGACTTGACTACTGA | |
| **AD76_4_G_for** | 5'Cy5-TCAGTAGTCAAGTCAGTGGTgAGGTCAGGACAGGAGTCAG | 40 bp EMSA probe with change in 4th position of BREX site |

| | | |
|---|---|---|
| **AD77_4_G_rev** | CTGACTCCTGTCCTGACCTcACCACTGACTTGACTACTGA | |
| **AD78_40_bp_scrambled_for** | 5’Cy5-TCAGTAGTCAAGTCAGTtagggaGTCAGGACAGGAGTCAG | 40 bp EMSA probe with change in 4th position of BREX site 40 bp EMSA probe with scrambled BREX site |
| **AD79_40_bp_scrambled_rev** | CTGACTCCTGTCCTGACtccctaACTGACTTGACTACTGA | |
| **Probe_2_s _F** | 5’Cy5-AGTCAGTAGTGAGGTCAGGAATGAGTCAGTCTCACTGTCAGGA | “scrambled” – 43 bp control probe with scrambled BREX site |
| **Probe_2_s _R** | TCCTGACAGTGAGACTGACTCATTCCTGACCTCACTACTGACT | |
| **Probe_2_BREX1_F** | 5’Cy5-AGTCAGTGGTAAGGTCAGGAATGAGTCAGTTGGCAAGTCAGGA | “1 BREX site” – 43 bp probe with one BREX site |
| **Probe_2_BREX1_R** | TCCTGACTTGCCAACTGACTCATTCCTGACCTTACCACTGACT | |
| **Probe_2_BREX2_hh_F** | 5’Cy5-AGTCAGTGGTAAGGTCAGGAATGAGTCAGTCTTACCGTCAGGA | “2 BREX sites” – 43 bp probe with two BREX sites in head-to-head orientation |
| **Probe_2_BREX2_hh_R** | TCCTGACGGTAAGACTGACTCATTCCTGACCTTACCACTGACT | |
| **Probe_2_BREX2_ht_F** | 5’Cy5-AGTCAGTGGTAAGGTCAGGAATGAGTCAGTGGTAAGGTCAGGA | “Head-to-Tail” – 43 bp probe with two BREX sites in head-to-tail orientation |
| **Probe_2_BREX2_ht_R** | TCCTGACCTTACCACTGACTCATTCCTGACCTTACCACTGACT | |
| **Probe_1_BREX_F** | 5’Cy5-AGTCAGTGGTAAGGTCAGGA | 20 bp probe with BREX site in the middle |
| **Probe_1_BREX_R** | TCCTGACCTTACCACTGACT | |
| **Probe_30_BREX_F** | 5’Cy5-AGTCAAGTCAGTGGTAAGGTCAGGACAGGA | 30 bp probe with BREX site in the middle |
| **Probe_30_BREX_R** | TCCTGTCCTGACCTTACCACTGACTTGACT | |
| **Probe_40_BREX_F** | 5’Cy5-TCAGTAGTCAAGTCAGTGGTAAGGTCAGGACAGGAGTCAG | 40 bp probe with BREX site in the middle |
| **Probe_40_BREX_R** | CTGACTCCTGTCCTGACCTTACCACTGACTTGACTACTGA | |
| **Probe_50_BREX_F** | 5’Cy5-AGTCATCAGTAGTCAAGTCAGTGGTAAGGTCAGGACAGGAGTCAGCAGGA | 50 bp probe with BREX site in the middle |
| **Probe_50_BREX_R** | TCCTGCTGACTCCTGTCCTGACCTTACCACTGACTTGACTACTGATGACT | |
| **Probes for BLI (reverse primers are the same as in EMSA)** | | |
| **AD25_TRB2040** | 5’biotin-TCAGTAGTCAAGTCAGTGGTAAGGTCAGGACAGGAGTCAG | 40 bp BLI probe with BREX site in the middle |
| **AD26_TRB2039** | CTGACTCCTGTCCTGACCTTACCACTGACTTGACTACTGA | |

| | | |
|---|---|---|
| **AD27_polyC_40_F** | 5'biotin-TCAGTAGTCAAGTCAGTCCCCCCGTCAGGACAGGAGTCAG | 40 bp BLI probe with polyC on place of BREX site |
| **AD28_polyC_40_R** | CTGACTCCTGTCCTGACGGGGGGACTGACTTGACTACTGA | |
| **AD35_BREX_20_F** | 5'biotin-AGTCAGT<u>GGTAAG</u>GTCAGGA | 20 bp BLI probe with BREX site in the middle |
| **AD36_BREX_20_R** | TCCTGACCTTACCACTGACT | |
| **AD37_polyC_20_F** | 5'biotin-AGTCAGTCCCCCCGTCAGGA | 20 bp BLI probe with polyC on place of BREX site |
| **AD38_polyC_20_R** | TCCTGACGGGGGGACTGACT | |

**Supplementary Table S4. EM data collection and refinement statistics**

| | **BrxX$_{apo}$**<br>PDB: 9EXH | **BrxX:Ocr (BrxX$_2$:Ocr$_2$)**<br>PDB: 9EX7 | **BrxX:DNA (43bp, H2T)**<br>PDB: 9EWZ |
|---|---|---|---|
| **Data collection** | | | |
| Microscope | TFS Krios | TFS Krios | TFS Krios |
| Magnification | 120 000 X | 120 000X | 165 000 X |
| Voltage (kV) | 300 | 300 | 300 |
| Electron dose ($e^-$/ Å$^{-2}$) | 35.1 | 35.1 | 50.5 |
| Detector | FEI Falcon IV (4k x 4k) | FEI Falcon IV (4k x 4k) | FEI Falcon IV (4k x 4k) |
| Defocus range (-µm) | 3 – 0.9 | 3 – 0.9 | 2.4 – 0.7 |
| Pixel size (Å) | 0.68 | 0.68 | 0.74 |
| Symmetry imposed | C1 | C1 | C1 |
| Micrographs (no.) | 5 100 | 5 100 | 11 152 |
| Initial particle images (no.) | 396 923 | 396 923 | 610 100 |
| Final particle images (no.) | 24 284 | 94 764 | 216 233 |
| Global map resolution (Å) | 4.13 | 2.84 | 2.22 |
| Fourier shell correlation (FSC) threshold | 0.143 | 0.143 | 0.143 |
| | | | |
| **Refinement Statistics** | | | |
| Model Resolution (Å) | 4.0 (4.1) | 2.91 (3.44) | 2.21 (2.54) |
| FSC Threshold | 0.143 (0.5) | 0.143 (0.5) | 0.143 (0.5) |
| Map sharpening *B* factor (Å$^2$) | -- | -- | -- |
| **Model Composition** | | | |
| Non-hydrogen atoms | 9 728 | 21 099 | 10 789 |
| Protein residues | 1205 | 1205 | 1205 |
| Nucleotides | 0 | 0 | 50 |
| Ligands | 0 | 2 | 2 |
| ***B* factors (Å$^2$)** | | | |
| Protein | 183.81 | 132.03 | 93.98 |
| Nucleotides | 0 | 0 | 80.39 |
| Ligands | 0 | 96.36 | 76.84 |
| **R.M.S deviations** | | | |
| Bond lengths (Å) | 0.002 | 0.003 | 0.002 |
| Bond angles (°) | 0.414 | 0.499 | 0.470 |
| **Validation** | | | |
| MolProbity score | 1.67 | 1.98 | 1.40 |
| Clashscore | 7.78 | 4.78 | 3.54 |
| **Ramachandran statistics** | | | |
| Favored (%) | 96.34 | 95.24 | 96.26 |
| Allowed (%) | 3.66 | 4.76 | 3.74 |
| Outliers (%) | 0.00 | 0.00 | 0.00 |

**Supplementary Table S5. Structural alignments of MTD and TRD of BrxX (DNA-bound) with homologous enzymes, and with an Ocr-bound structure.** Shown are outputs from ChimeraX *matchmaker* programme.

| | | **CamA** | | **MmeI** | | **DrdV** | | **HsdM** | | **Ocr-Bound BrxX** | | | |
|---|---|---|---|---|---|---|---|---|---|---|---|---|---|
| | | MTD | TRD | MTD | TRD | MTD | TRD | MTD | TRD | NTD | MTD | TRD | CTD |
| **DNA-bound BrxX** | RMSD (Pruned Atom Pairs) | 1.056 (154) | 1.091 (72) | 1.113 (103) | 1.323 (17) | 0.988 (128) | 1.463 (6) | 1.101 (101) | 0.239 (9) | 0.891 (209) | 0.888 (392) | 0.838 (173) | 0.854 (339) |
| | RMSD (All Pairs) | 13.295 (271) | 5.160 (164) | 11.816 (296) | 21.879 (159) | 8.358 (274) | 22.205 (70) | 12.980 (241) | 16.905 (40) | 0.901 (210) | 1.324 (416) | 1.085 (187) | 1.570 (368) |

## SUPPLEMENTARY REFERENCES


1. Ashkenazy, H. *et al.* ConSurf 2016: an improved methodology to estimate and visualize evolutionary conservation in macromolecules. *Nucleic Acids Res* **44**, W344–W350 (2016).
2. Pettersen, E. F. *et al.* UCSF ChimeraX: Structure visualization for researchers, educators, and developers. *Protein science* **30**, 70–82 (2021).
3. Gordeeva, J. *et al.* BREX system of Escherichia coli distinguishes self from non-self by methylation of a specific DNA site. *Nucleic Acids Res* **47**, 253–265 (2019).
4. Isaev, A. *et al.* Phage T7 DNA mimic protein Ocr is a potent inhibitor of BREX defence. *Nucleic Acids Res* **48**, 5397–5406 (2020).

**Source Data for Figure 1C**

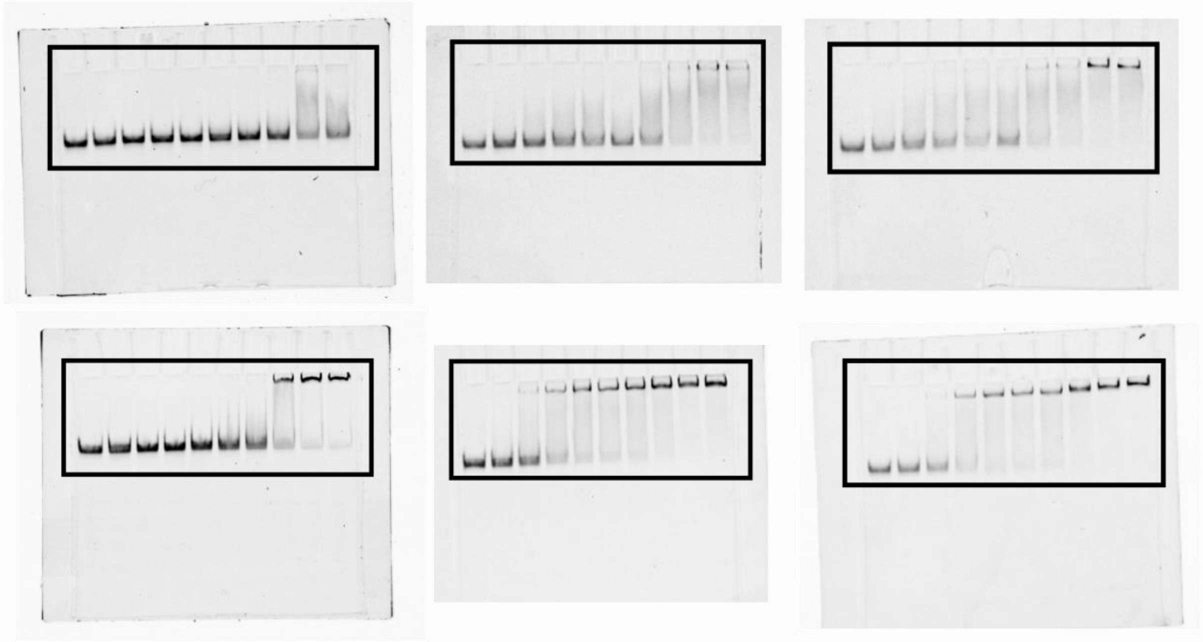

**Source Data for Figure 1F**

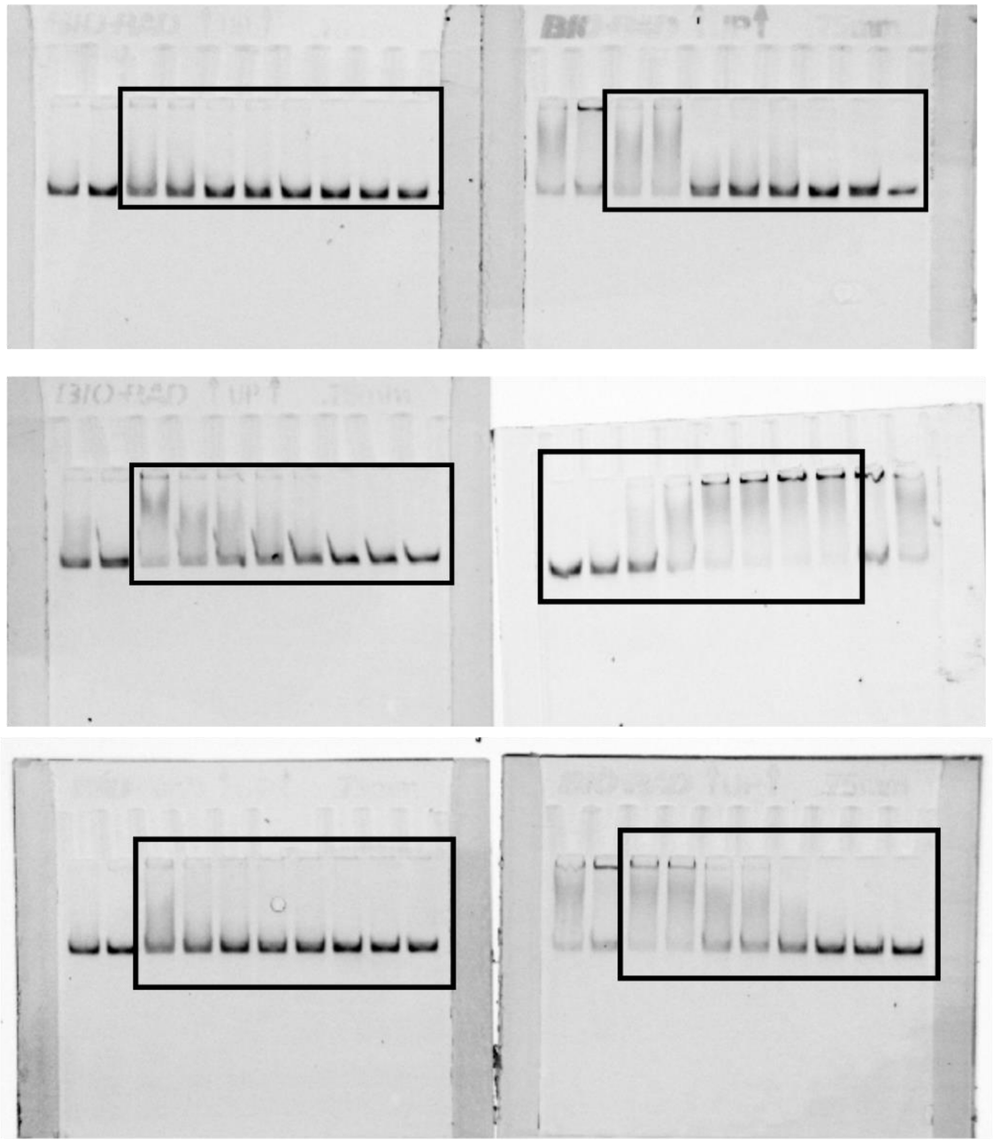

**Source Data for Figure 4A**

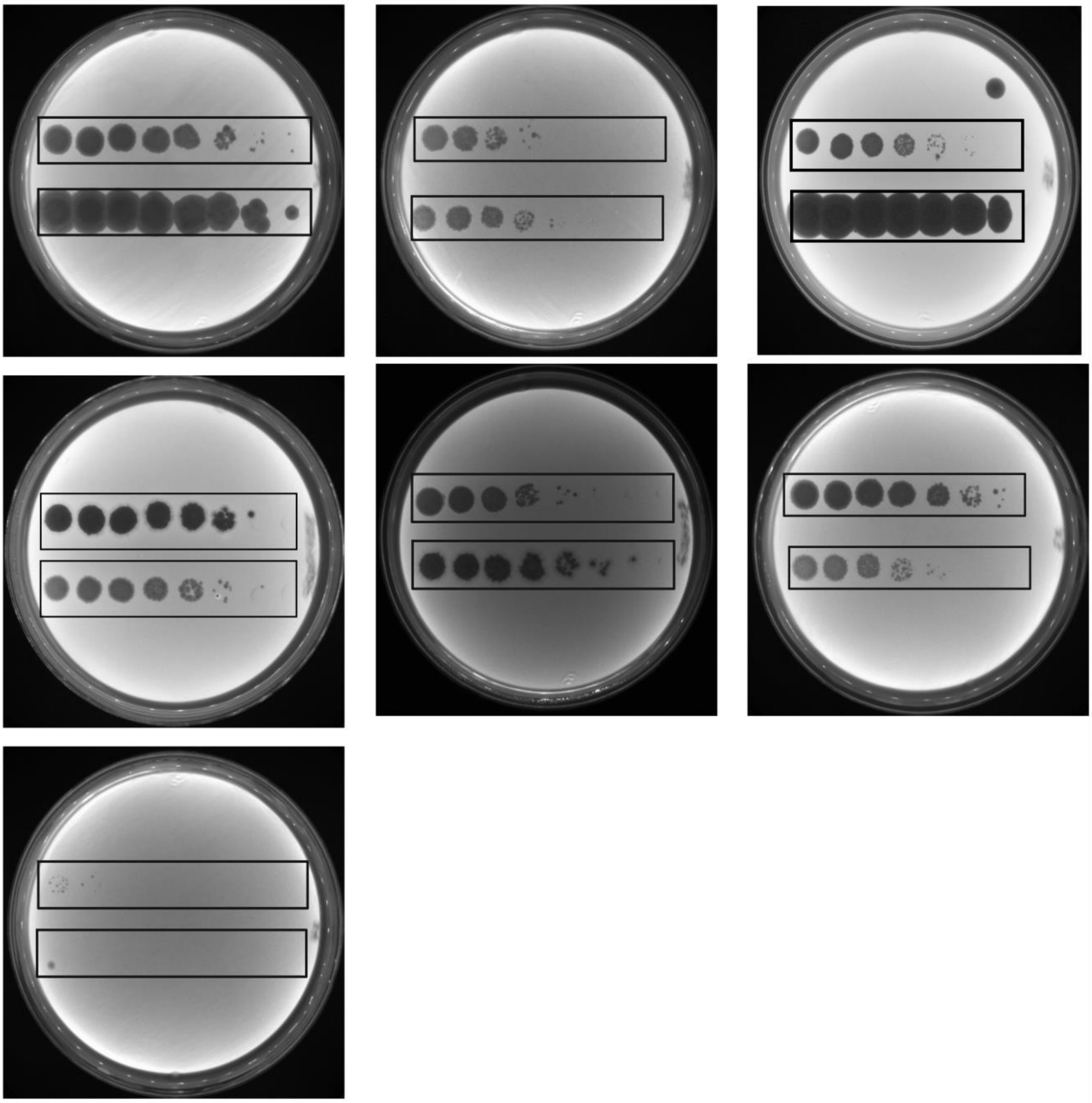

## Source Data for Figure 4B

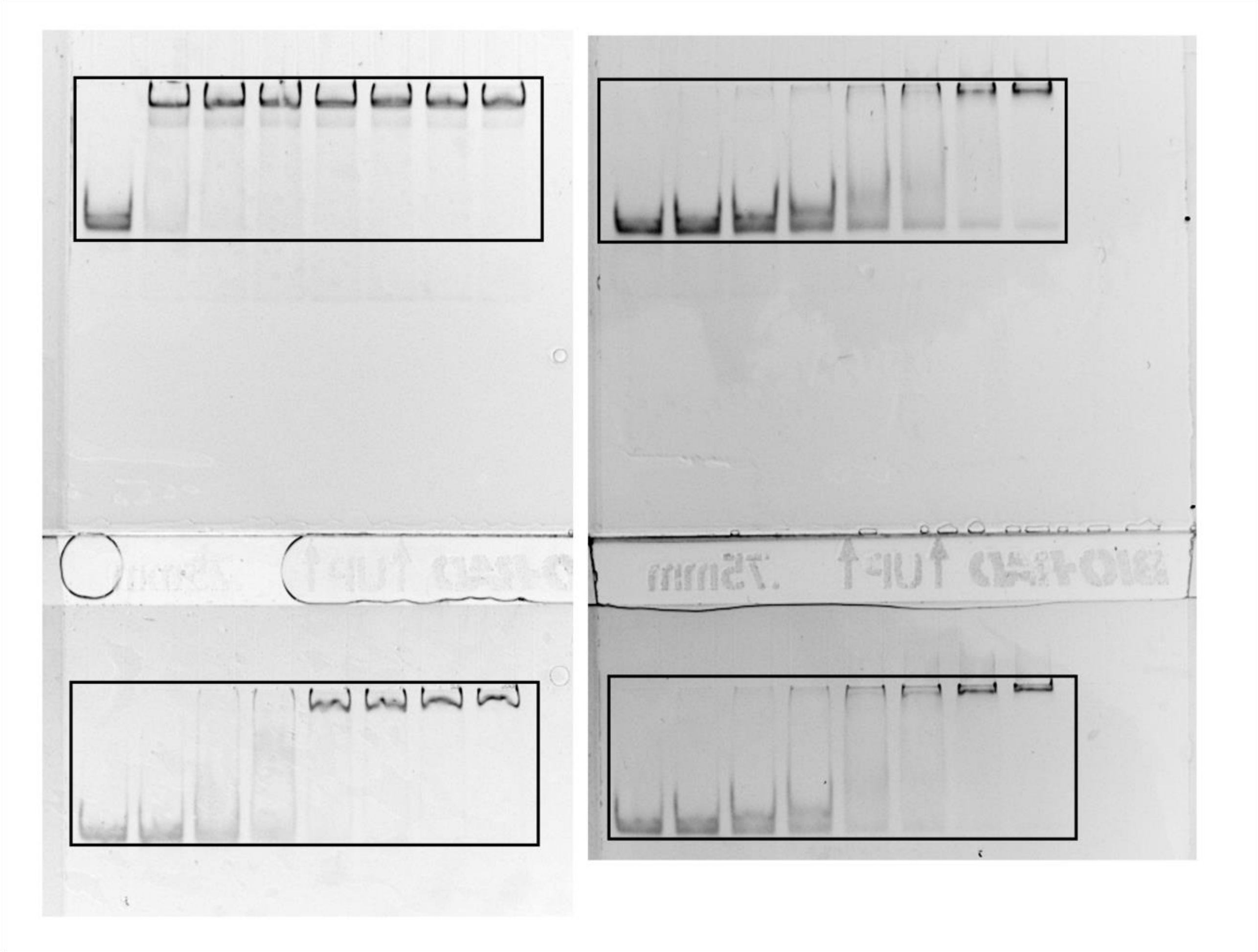

**Source Data for Figure 4D**

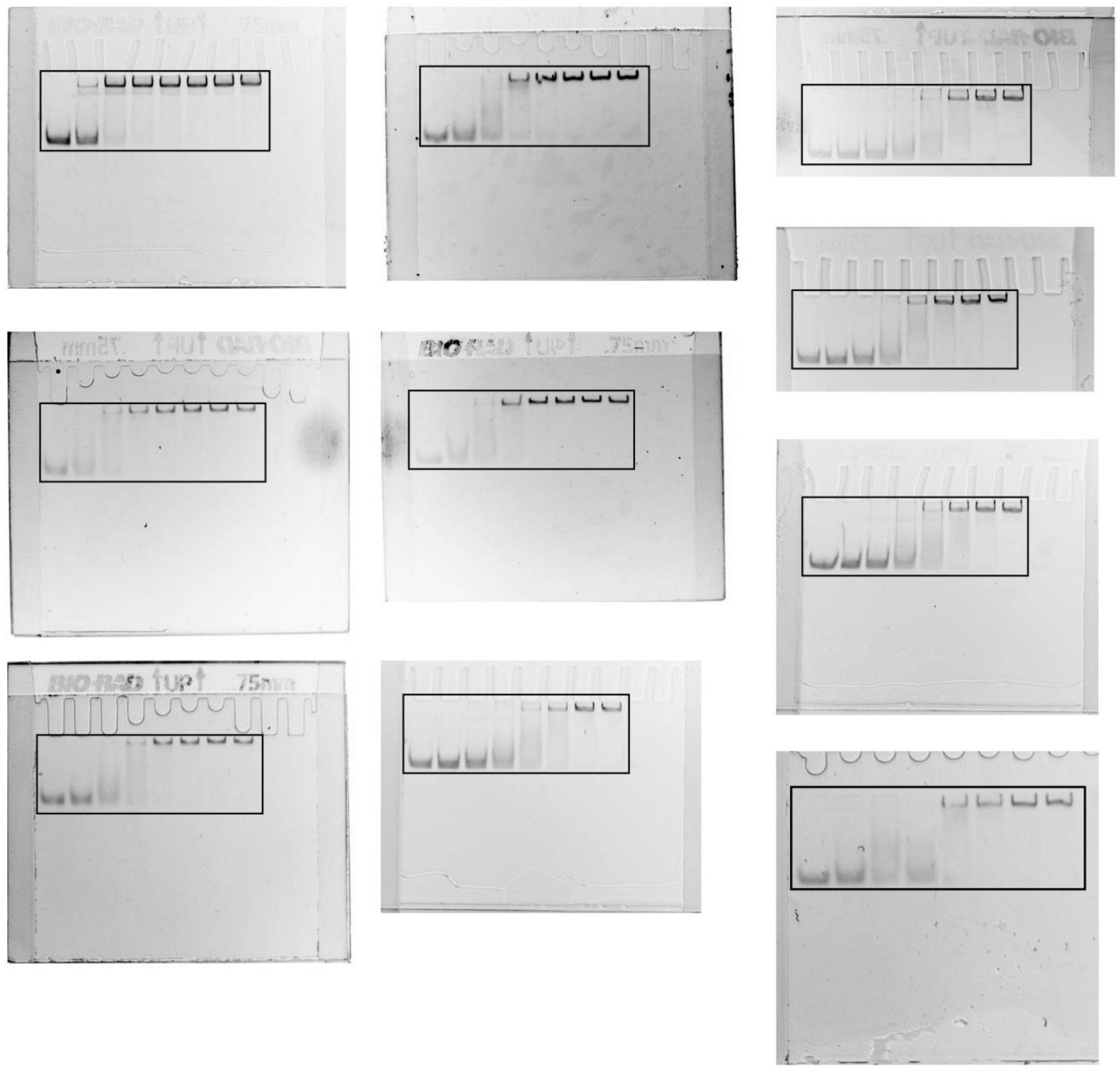

**Source Data for Figure 5B**

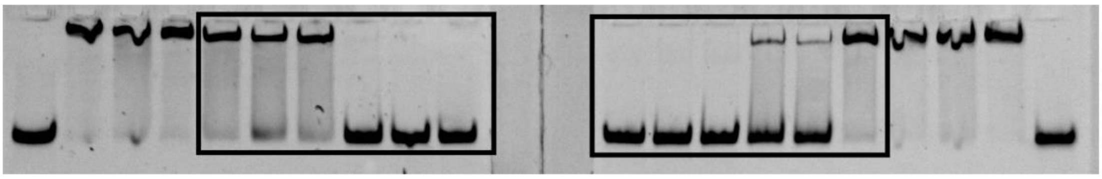

**Source Data for Figure 5C**

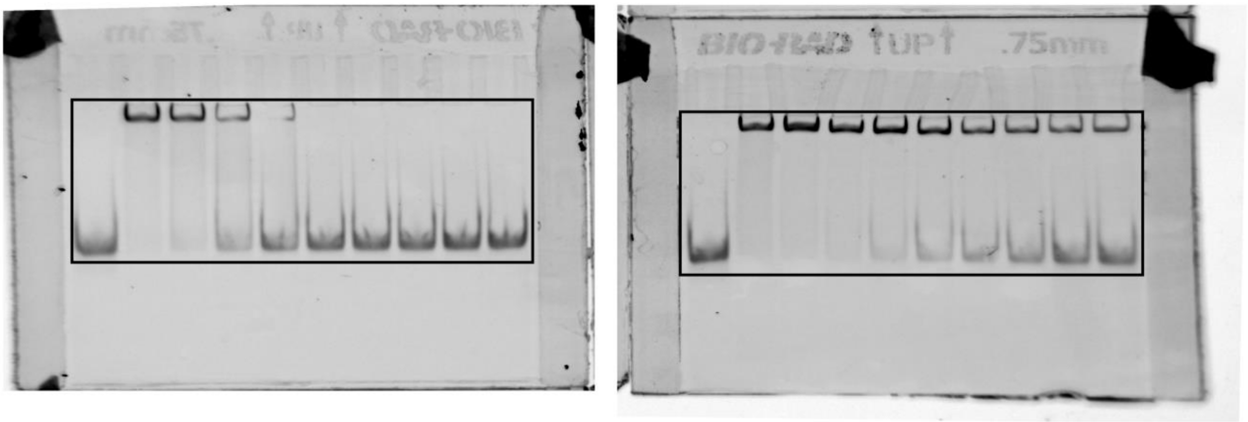

**Source Data for Figure 5I**

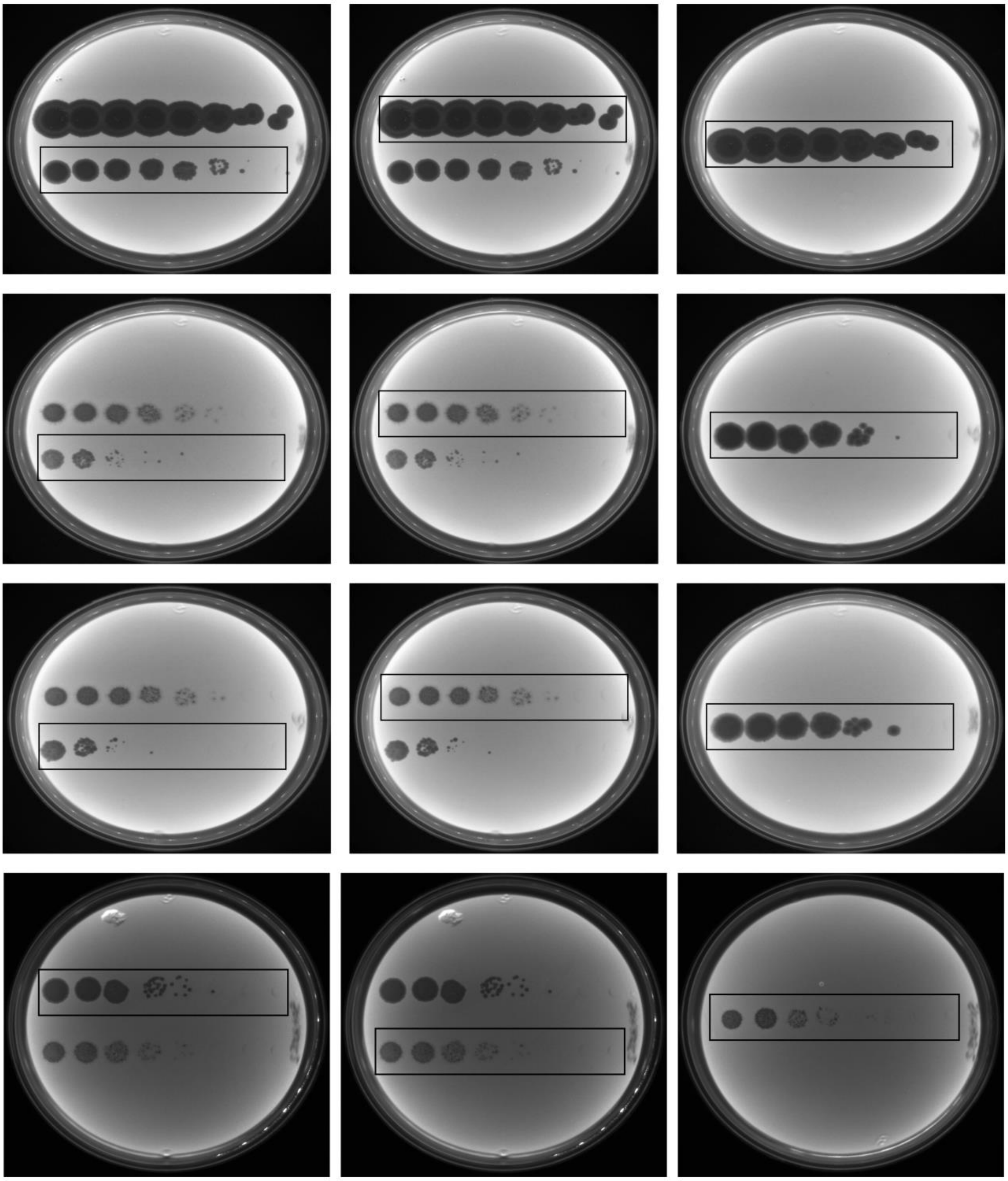

**Source Data for Figure 6C**

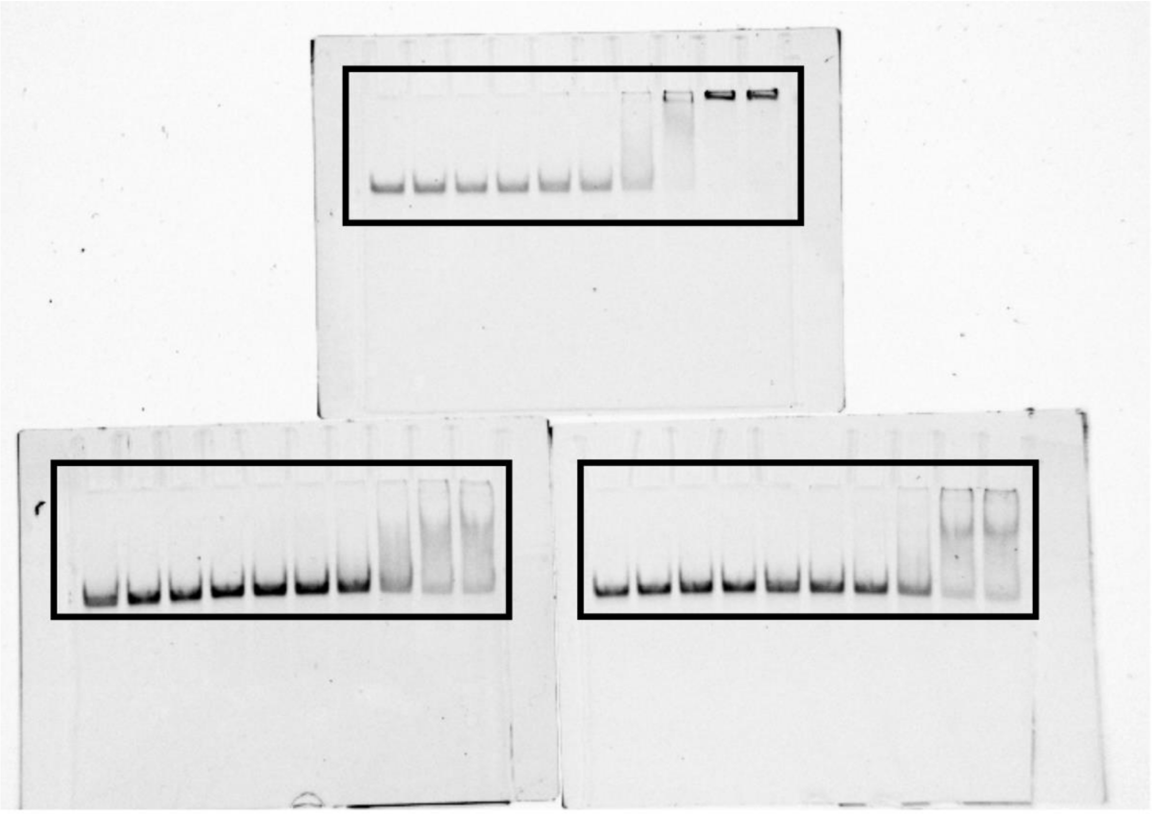

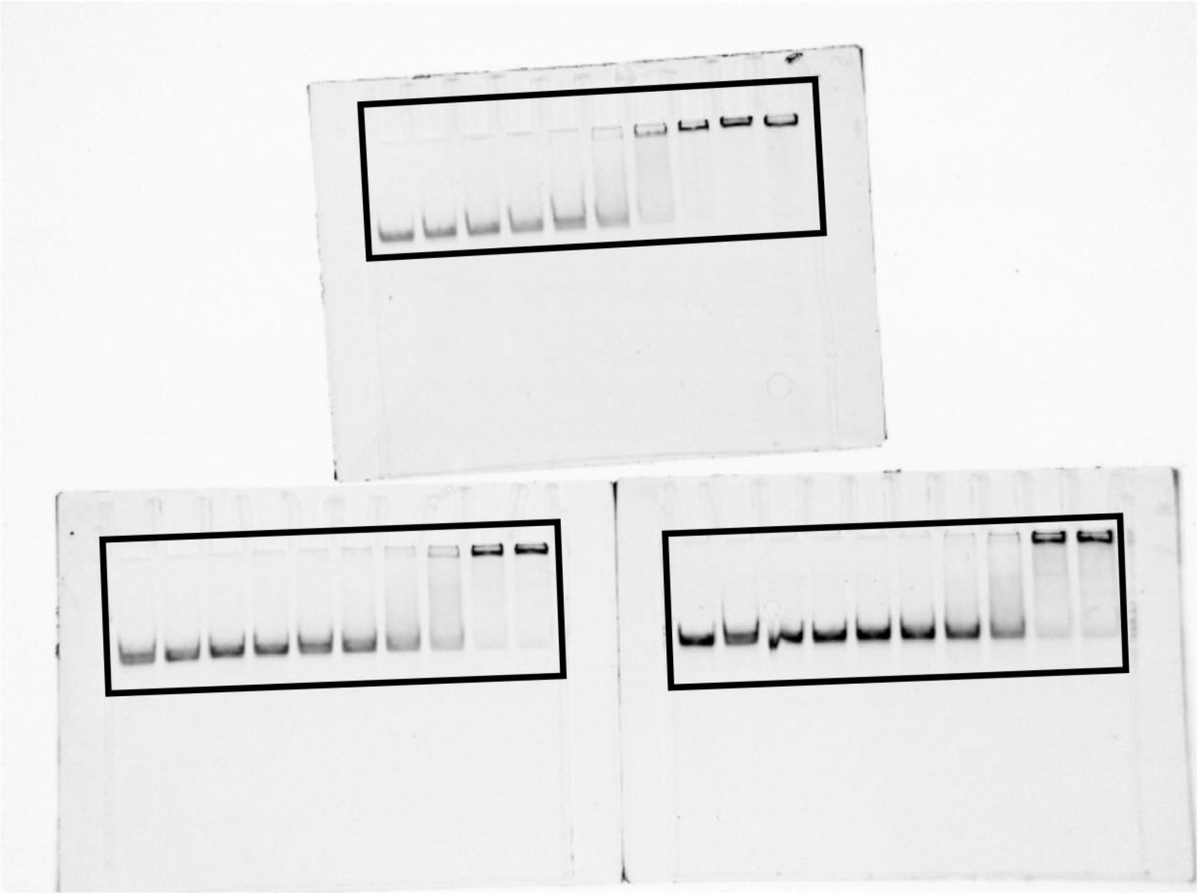

## Source Data for Figure 6D

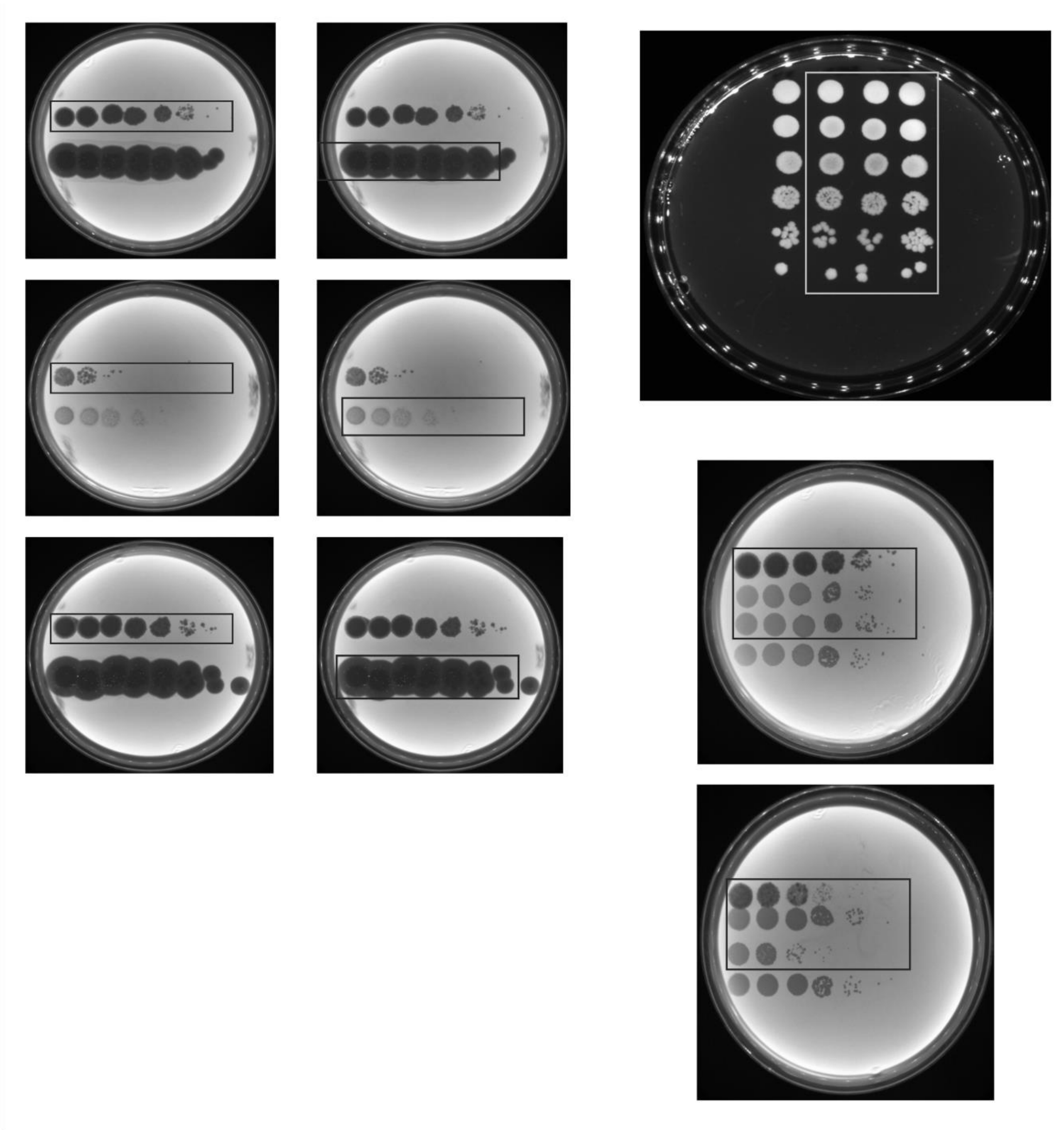

**Source Data for Figure 6E**

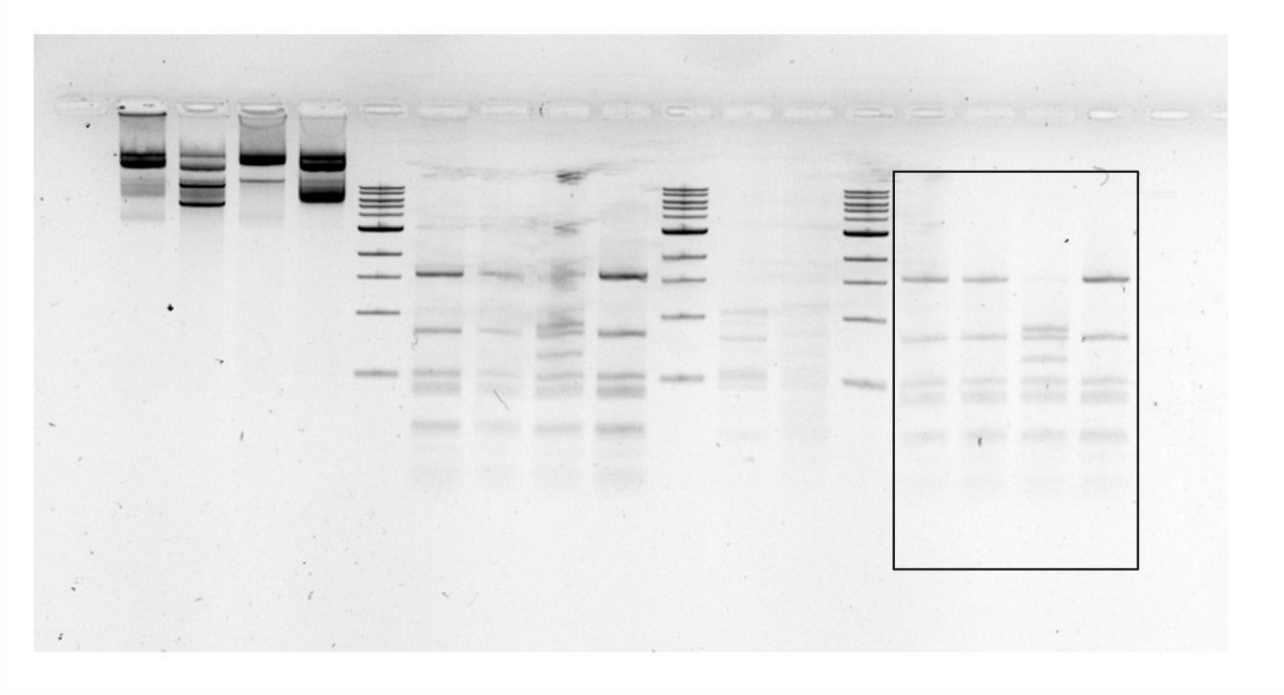

**Source Data for Figure 6F**

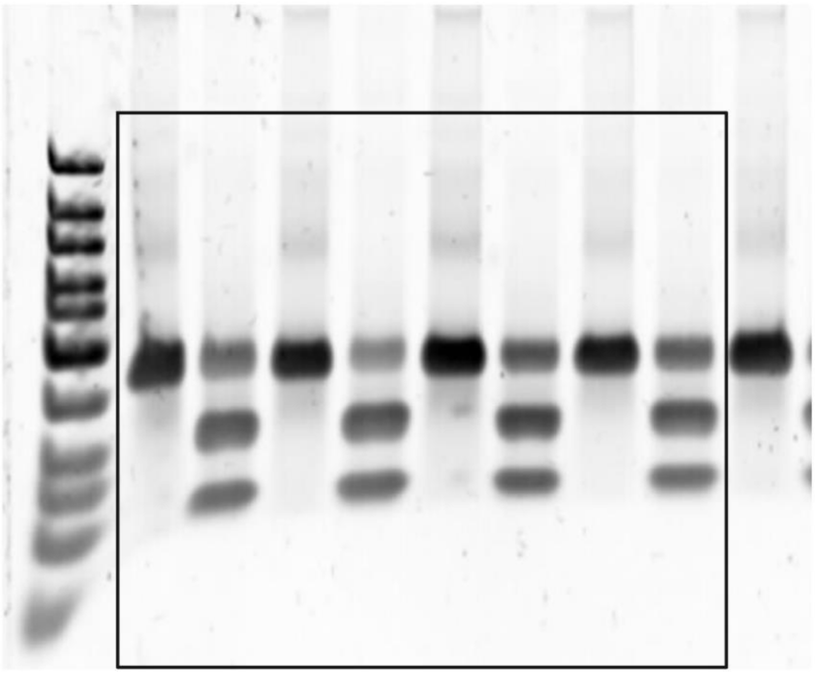

**Source Data for Figure 7B**

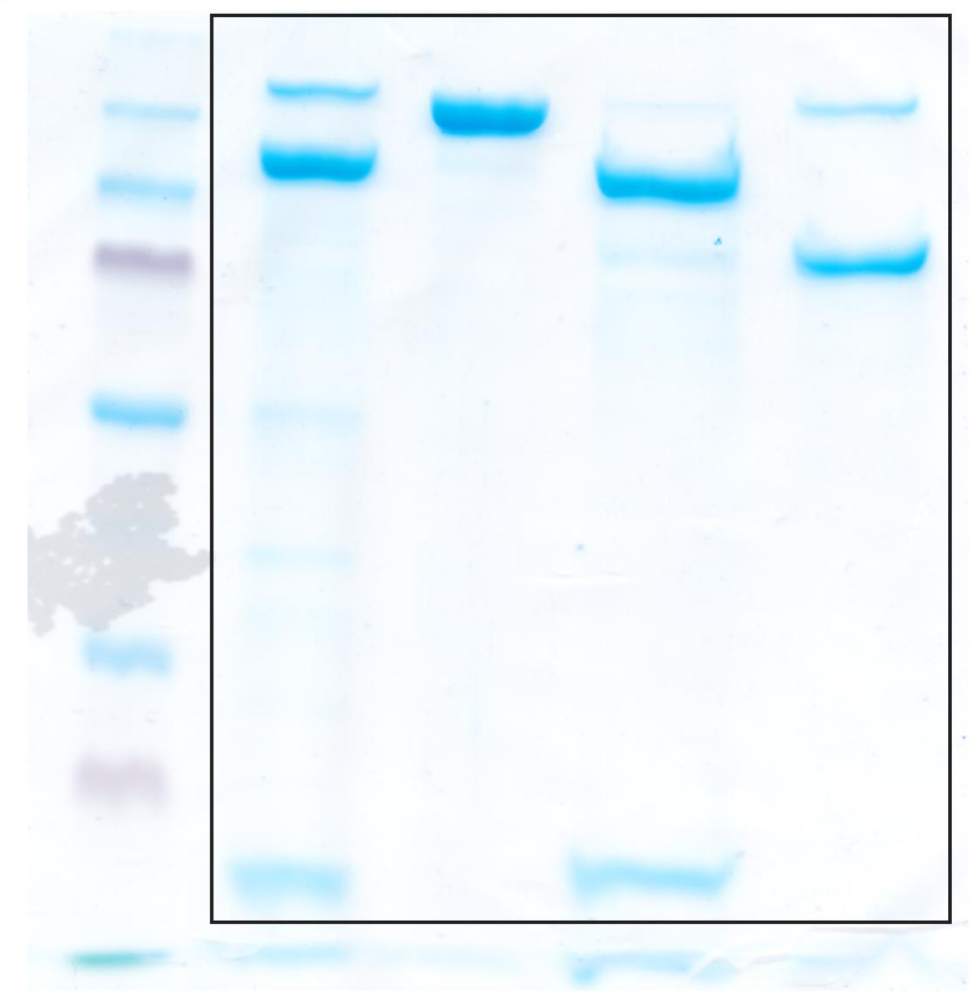

**Source Data for Figure 7C**

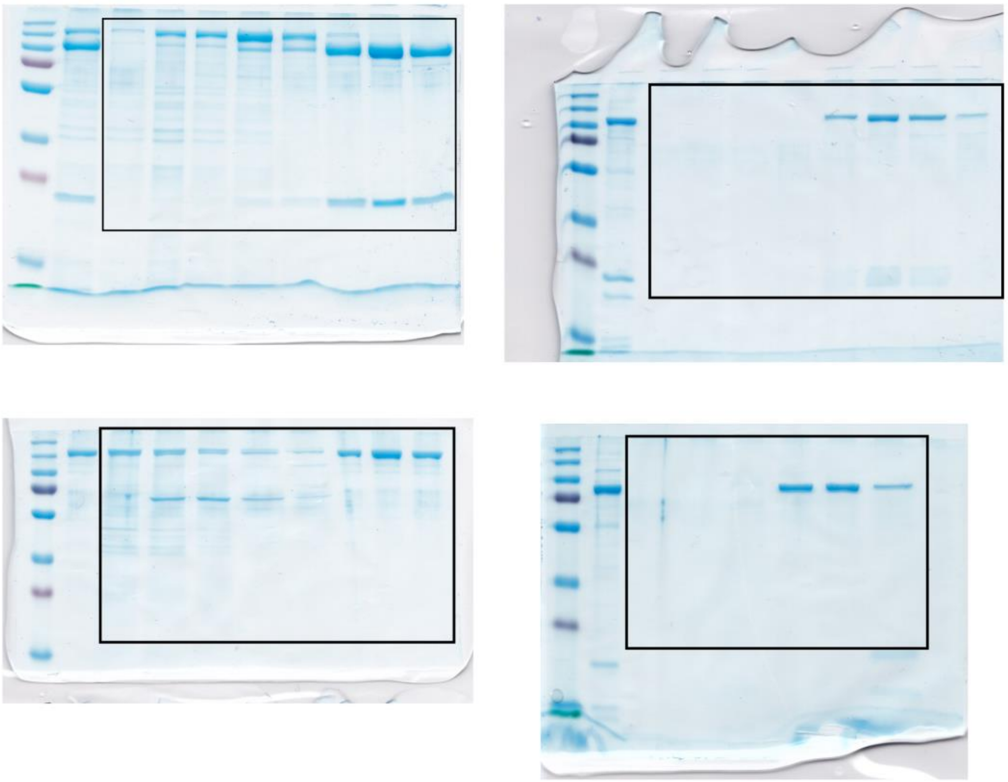

**Source Data for Figure 7F**

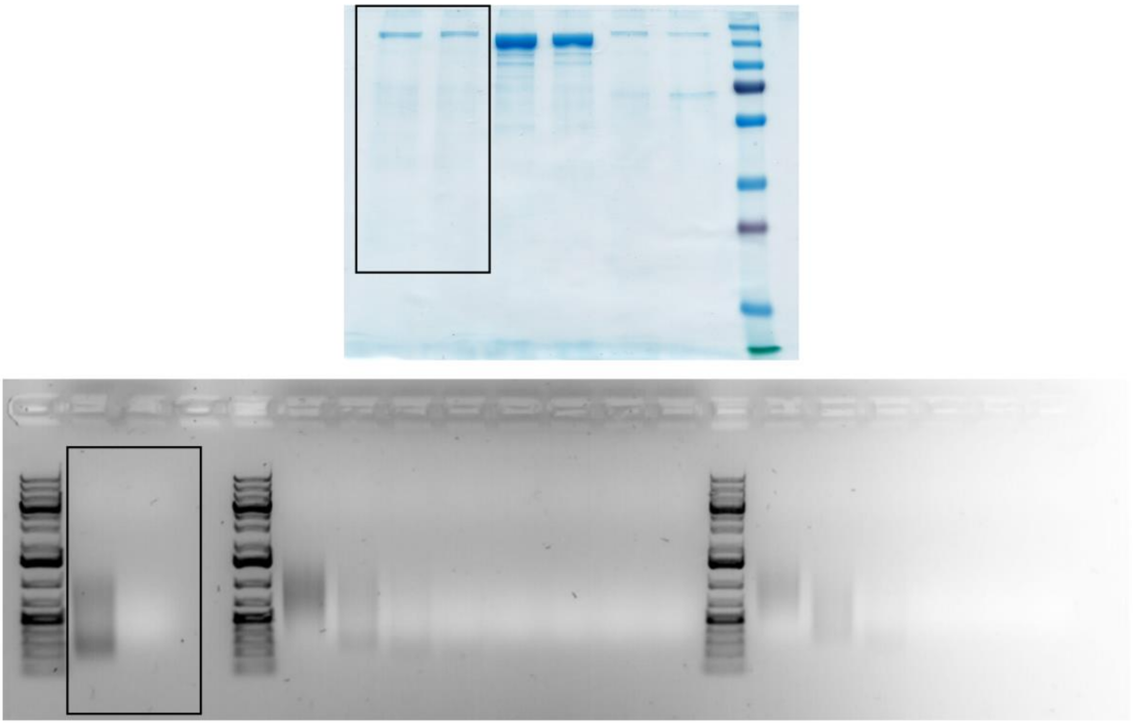

**Source Data for Extended Data Figure 1**

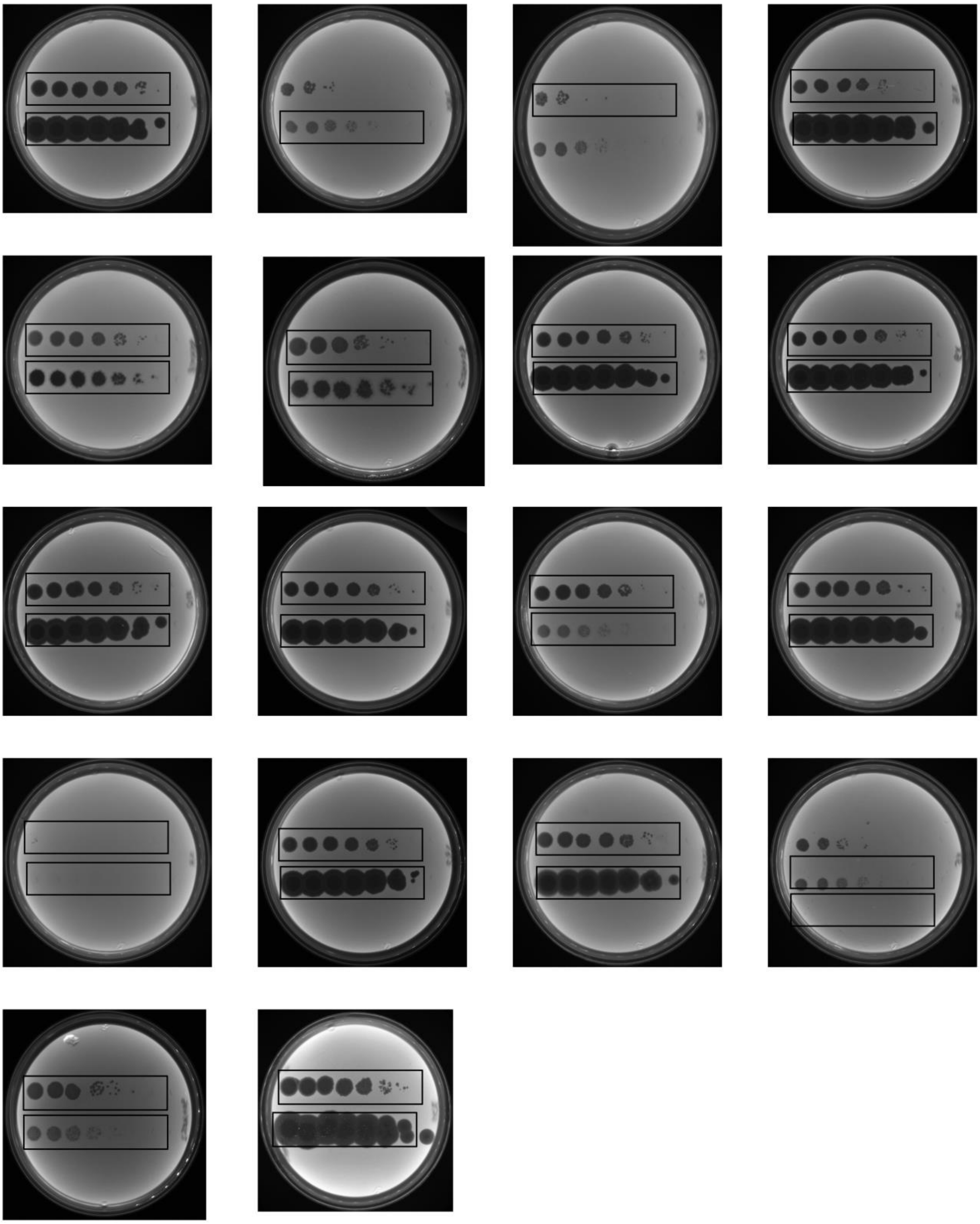

## Source Data for Supplementary Figure 1A

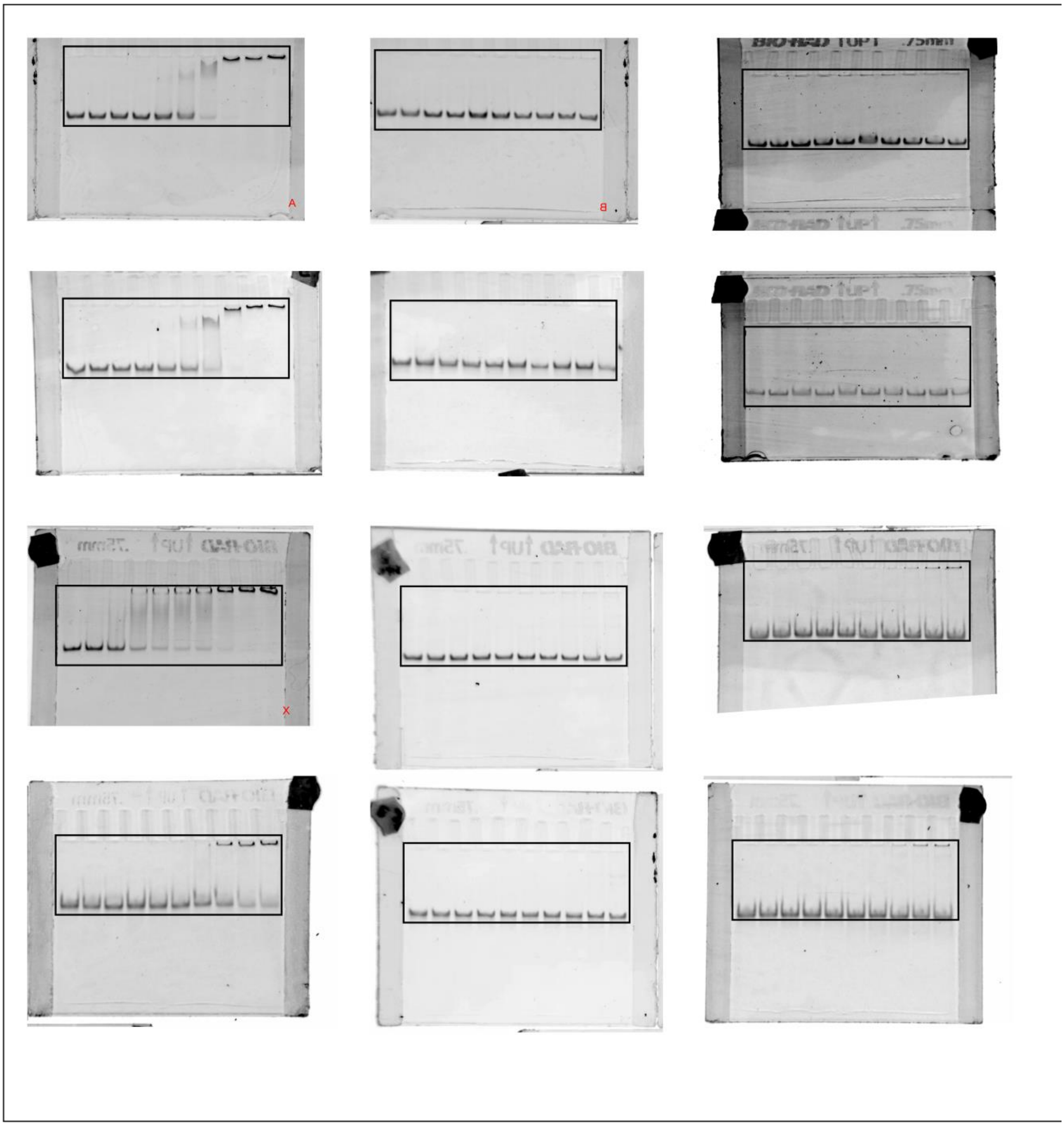

**Source Data for Supplementary Figure 1B**

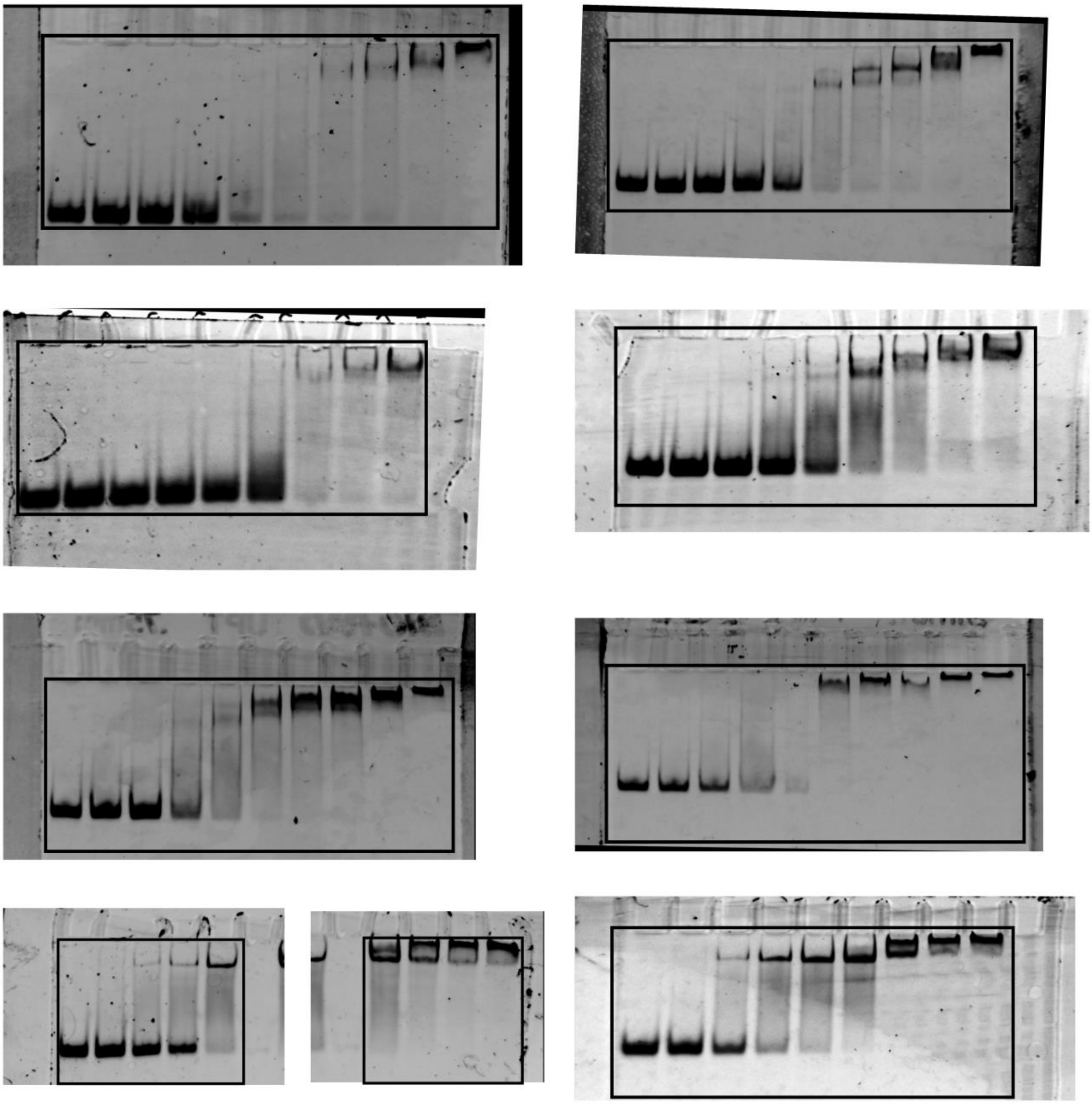

**Source Data for Supplementary Figure 1C**

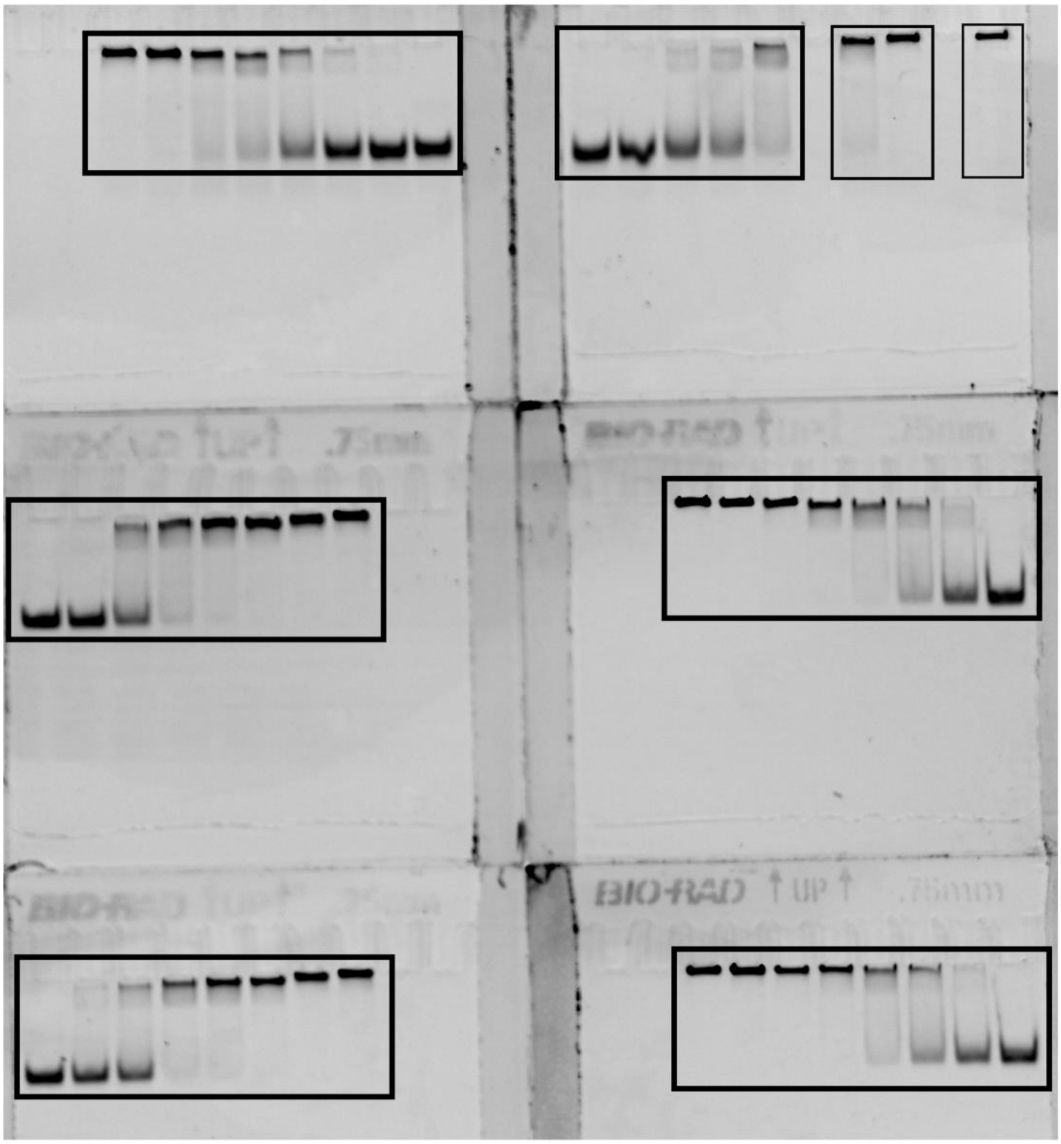

**Source Data for Supplementary Figure 3A**

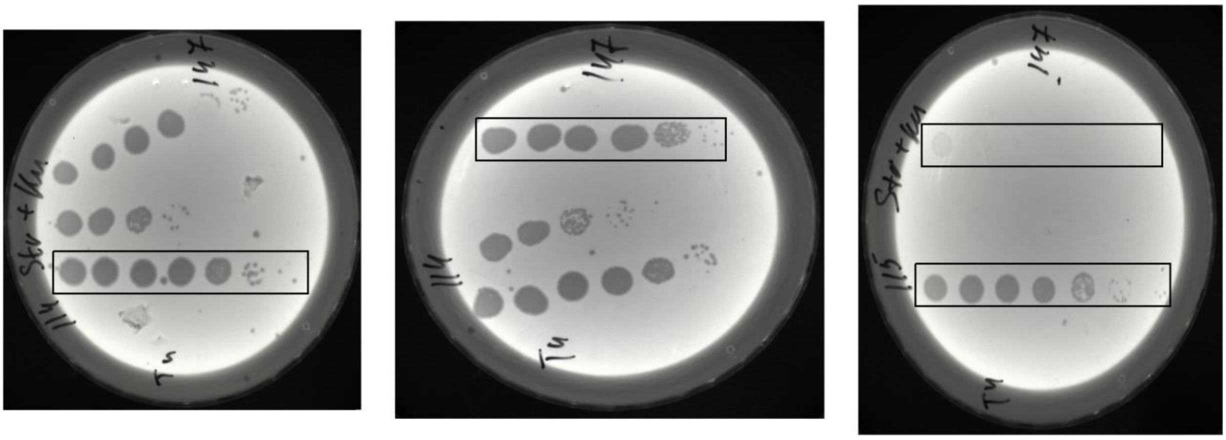

**Source Data for Supplementary Figure 3B**

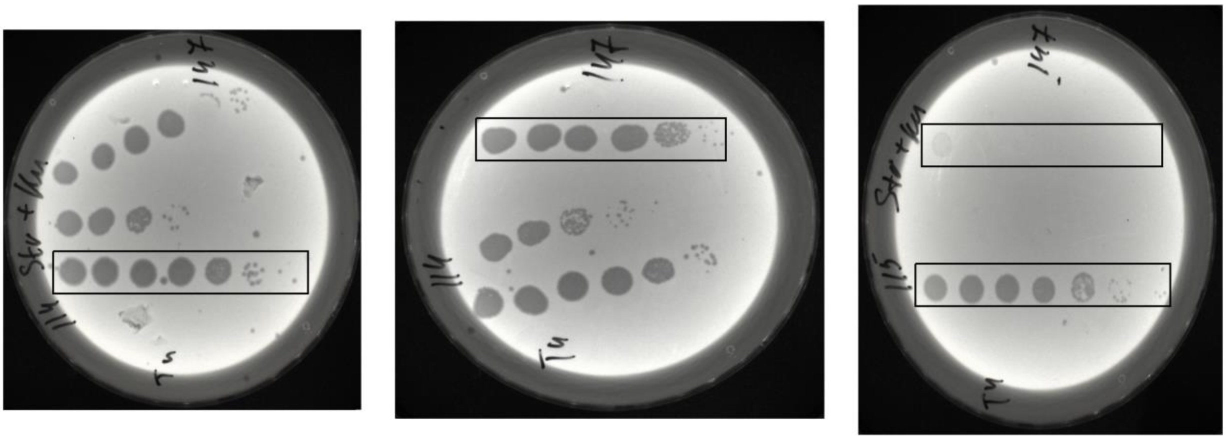

**Source Data for Supplementary Figure 3C**

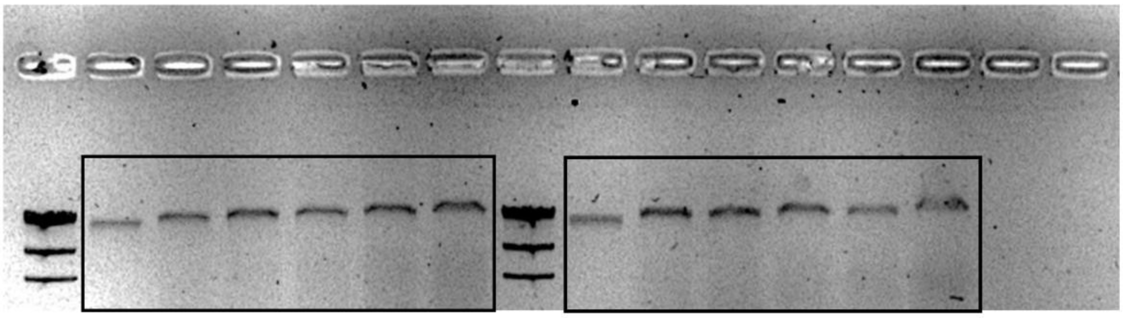

**Source Data for Supplementary Figure 9D**

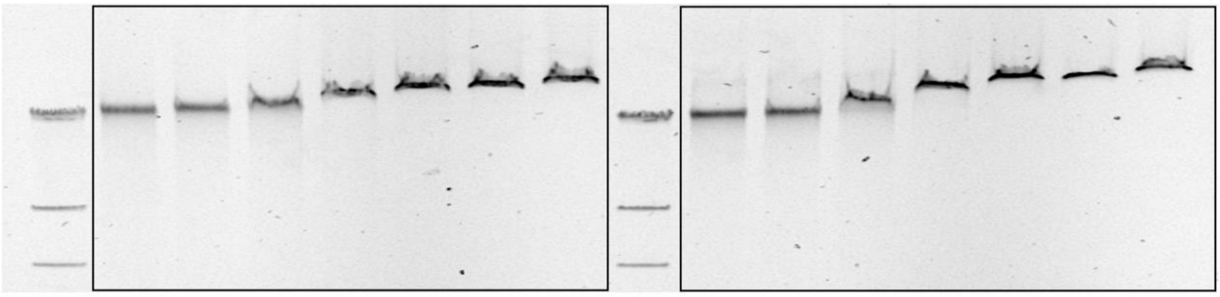

**Source Data for Supplementary Figure 9E**

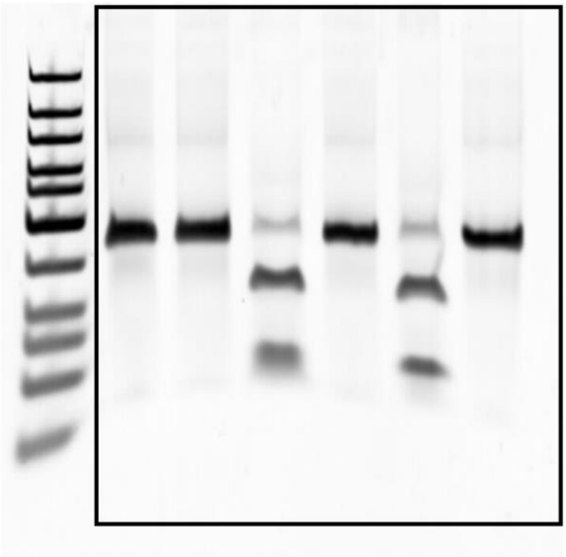

**Source Data for Supplementary Figure 9G**

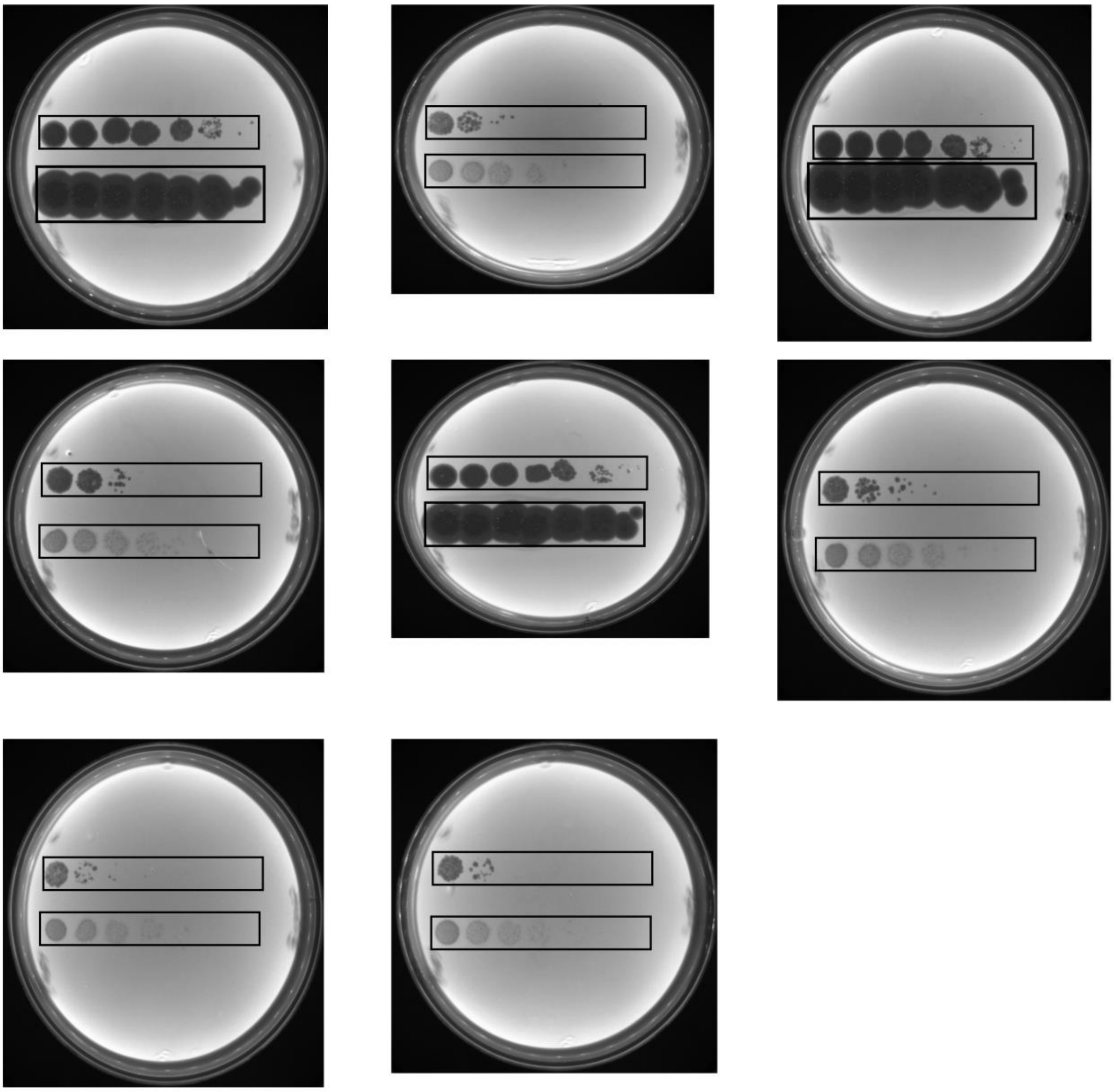

Supplement: Supplementary file 1 — Supplementary Information [file 41467_2025_57006_MOESM1_ESM.pdf]
